# Evolutionary lags in the primate brain size/body size relationship revisited

**DOI:** 10.1371/journal.pone.0351073

**Published:** 2026-07-01

**Authors:** R. I. M. Dunbar

**Affiliations:** Department of Experimental Psychology, University of Oxford, South Parks Road, Oxford, United Kingdom; Universita Campus Bio-Medico di Roma, ITALY

## Abstract

The original brain lag hypothesis proposed that primate brain evolution depended on spare energy derivative of savings of scale enabled by first increasing body size. However, in a seminal analysis, Deaner & Nunn concluded that there was no evidence for a brain lag. I revisit their analysis and show that using statistically more appropriate analyses and updated data yields a significant brain lag effect. However, contrary to the original brain lag hypothesis, the brain/body ratio does not converge back on the allometric regression line, but continues to evolve beyond it. Increases in brain size are correlated with exploiting large group size rather than large body size as the principal defence against predation risk, with significant growth in brain size (but not body size) only being possible if species adopt a more frugivorous diet. I then use these findings to show that hominins followed a similar trajectory from an australopithecine baseline on the relevant allometric regression. In sum, the brain lag effect is much more complicated than the original hypothesis proposed, with a distinctive switch from emphasising body size to brain size (hence group size) as a solution to ecological challenges.

## Introduction

Ever since Jerison’s [1] seminal analyses, it has been known that, across mammals in general and primates in particular, brain size is an allometric power function of body mass, with different orders forming clearly distinct grades. An early suggestion was that much of the internal variance around the order-specific allometric regression line, especially in large-brained primates, was a consequence of a lag effect in which body size changes first, with brain mass taking time to catch up with the changes in body mass [1–7].

This assumption was based mainly on the fact that body size is relatively labile, and can vary considerably within species as a function of local environmental conditions [8–9], whereas the complex interconnectivity of brain systems means that it is likely to take longer to bring together the necessary genetic changes without disrupting functional neural systems. The fact that changes in body size clearly outstripped changes in brain size during domestication [10–12] (notwithstanding rather questionable claims to the contrary by [13]) lends support to this. Given this, it was assumed that, with enough time, brain and body size would inevitably converge back on the common allometric regression line as brain size expanded to absorb the additional energy made available by a larger body mass through the efficiencies provided by Kleiber’s Law [14–16]. The implication is that the allometric regression acts as an attractor to which the system naturally gravitates.

Deaner & Nunn [17] developed a particularly innovative method for testing the brain lag hypothesis that involved plotting the residuals of the phylogenetic contrasts in brain mass regressed on the contrasts in body mass against date of divergence. Since the original hypothesis assumed that body mass will always change first, the lag will necessarily be directional. Hence, they argued, the magnitude of the signed residual from the brain-to-body size allometric relationship should be positively correlated with divergence date when body mass contrasts are constrained to be positive: ‘young’ nodes (i.e., recent speciation events where body size has changed but not brain size) should consist of species pairs with strongly negative residuals (smaller brains relative to body size), whereas nodes with deeper divergence dates that have had more time to readjust will approach the allometric line (where residuals equal 0). They found that, for a sample of primates, there was no correlation between the two variables, either for males (r=-0.20, p=0.38) or for females (r=0.15, p=0.480), or when controlling for species’ typical circadian rhythms.

This led them to conclude that there was no evidence for the lag hypothesis. By implication, this implies that brain and body size change simultaneously under the same environmental pressure, just as is the case for brain size and group size in primates (but not other mammals) [18]. Because these results seemed conclusive, the hypothesis lost traction and has received little attention in the literature since. However, a number of recent developments in our understanding of primate evolutionary biology have raised the possibility that the original Deaner-Nunn result might be subject to several sources of confound. I identify four such issues that were not appreciated at the time of the original analysis.

One is the fact that, while most comparative studies have adopted a simple one-cause/one-effect view [19–20], recent analyses have identified that grades are present in many relationships. The slope for the brain/body size regression, for example, differs across the different mammalian orders [21–22] and between families within orders [22]. Grades have turned out to be particularly prominent in the primate social brain data [23–26]. When grades are present in data, any analysis that places an overall regression through the data will inevitably fall foul of the Yule-Simpson Paradox (a version of the Ecological Fallacy). Because of the way OLS regressions are computed, this will inevitably lower, and in some cases even reverse, the slope of the regression [27–28]. On a mouse-to-elephant scale, this may not matter, but on smaller taxonomic scales it can lead to quite misleading results [28]. This problem was discussed at some length during the early days of comparative analyses [29–32], but seems largely to have been forgotten. More importantly, grades may indicate that different groups of species respond in different ways, depending on the ecological drivers of brain and body size evolution and the ease with which genetic changes can be implemented.

Second, Deaner & Nunn’s analysis assumed that brain size is mainly a function of body size, with the two being genetically linked. But if brain size and body size are genetically uncoupled, as they appear to be in primates [2], there is no principled reason why body size should always have to change first or why brain size should subsequently have to “catch up” with body size (see also [20]). That brain and body mass are not yoked in close genetic linkage is confirmed by breeding experiments showing that they can undergo independent selection, at least in rodents [33]. Lande [2] examined the brain-to-body size relationship across mammals in order to evaluate the evolutionary coupling between these traits. He argued that the genetic correlation between brain and body size in primates is unusually weak compared to other mammalian orders. More importantly, he found that the variance in the primate allometric relationship increases with body size, suggesting that the two become progressively decoupled as body size increases [20,34,35].

Third, Deaner & Nunn reported that there was no correlation between their index of residual brain size and social group size (primates’ principal anti-predator strategy [25]). However, different species can, and do, pursue alternative anti-predator strategies, and this could explain the wide variation they observed in the residuals of brain size regressed on body size. Large body size and large group size have long been known to be alternative strategies that primates use (sometimes in combination) to reduce predation risk in order to occupy habitats subject to high predator densities [36–40]. In primates at least, group size has been shown to correlate strongly with brain size, both within (humans [41–48]; monkeys [49–51]) and between [18,19,23–26,28,52–54] species, because the cognitive demands imposed by increasing social group size in the kind of bonded societies characteristic of primates are neurologically very demanding [25,55–58]. (Note that the two studies claiming that brain size and group size are uncorrelated [59,60] unwittingly tested hypotheses about constraints, not hypotheses about selection effects. For details, see [28].)

The fourth issue is that the datings on the primate phylogeny have changed significantly in the last three decades as a result of improved molecular data. Deaner & Nunn [17] used divergence dates given by Purvis [61] which were based mainly on anatomical and fossil evidence. More recent estimates based on genetic coalescence, especially for the deeper divergences, might yield very different results.

The original brain lag hypothesis assumed that body size always changed first (causing initial residuals of brain on body mass to be negative). Deaner & Nunn therefore tested for a positive relationship between brain/body residuals and time since divergence. Strictly speaking (though they never tested for this), if the brain lag hypothesis is true, then residuals should converge back onto the allometric relationship and remain there until deflected once again by further changes in body size. But, if the residuals systematically project *beyond* the allometric regression (and do so increasingly with time post-divergence), this would be evidence against the lag hypothesis as originally conceived. More importantly, it would open up the rather more interesting possibility that a brain lag might allow species to catapult themselves onto a higher brain/body grade by exploiting the opportunities offered by Kleiber’s Law: larger body size creates greater savings of scale in metabolic energy demand, thereby allowing the surplus energy intake to be diverted into evolving an even larger brain.

I reanalyse the Deaner-Nunn data to determine whether a brain lag is evident if (a) we use updated phylogenetic divergence times and (b) we take grades into account if these are present. If there is evidence for a brain lag, this then raises several further questions about the direction of the lag, why the lag occurs (i.e., the environmental factors that select for a particular change) and how species balance the energy demands inevitably created by any such change. If we are to test the validity of the original analysis, it is essential to use exactly the same dataset and statistical methods as Deaner & Nunn [17] in order to be sure that any differences in findings are due to the analysis and not to differences in the data or methods. I then test (1) the hypothesis (derived from four independent path analyses [26,52,62]) that changes in brain or body size necessitated dietary changes to meet the energetic costs of the additional tissue (rather than being the driver of increases in brain and body size) and (2) the hypothesis that deviations from the allometric regression line are a response to high predation risk as the known principal driver of primate social evolution [36–40,]. Finally, the excellent fossil record for hominins offers a near-unique opportunity to test for a brain lag effect diachronically.

## Methods and materials

### Data

To test the brain lag hypothesis, Deaner & Nunn [17] suggested using standard CAIC contrasts to identify residuals from the common allometric brain/body size regression plotted against divergence time. For these purposes, contrasts were deliberately measured without reference to elapsed time so that they could use the date of the last common ancestor (the conventional Brownian motion control) as the independent variable in the analysis. In addition, they explicitly used only nodes that were tip comparisons (i.e., comparisons between living species) and avoided ancestral nodes at higher levels in the phylogeny because these can never be known with any precision.

Deaner & Nunn argued that it was crucial to use brain and body weights determined from the same anatomical specimen. The only dataset that met this criterion was the Stephan et al. [63] primate dataset. Using ECVs as a proxy for brain size on the grounds that it offers a larger sample size [64,65] was not considered an acceptable alternative because ECVs include intracranial spaces (the subdural spaces and ventricles) not occupied by actual brain tissue; as a result, ECV estimates are typically 5–10% larger than actual brain size, with the difference being larger in large-brained species [66]. ECV data invariably yield far more variable, less precise correlations with relevant behavioural and cognitive correlates than actual brain volumes do [25]. More problematically for present purposes, the body and brain size estimates in ECV datasets are often sourced from different populations. Because body size varies very considerably (by up to 50%) across populations of a species depending on local environmental conditions [8–9], brain:body size ratios will vary for reasons completely unrelated to evolutionary processes and we risk confounding phenotypic environmental effects with evolutionary genetic effects.

Although several primate brain datasets [25] have been produced since Stephan’s original, all are considerably smaller than Stephan’s; more importantly, they do not add many new genera. Rilling [67] strongly cautioned against mixing brain data based on different methods because of the different error variances involved. I therefore follow Deaner and Nunn’s advice, and use only the Stephan dataset since doing so allows me to directly compare my results with theirs. If we use another dataset and get different results, we will not know whether it was their data or the methodology that caused the difference.

Group size data for individual species are sourced from [68]. Diet data are sourced from [60], subject to a correction for *Macaca* given by [69]. For the reasons given by [25,28,70], actual predation rates are inappropriate for testing hypotheses about the historical impact of predation on the evolution of counterstrategies. It is the intrinsic environmental predation *risk* that drives the evolution of anti-predator strategies. Actual predation rates represent the predation risk that the animals have been unable to control by their evolved anti-predator strategies. Predation risk is a product of the local predator density, the availability of refuges (mainly trees), and body size [71,72]. Since body mass forms part of the brain/body mass residuals, I use an index of terrestriality as a proxy for predation risk (terrestrial habitats typically expose primates to higher predation risk with fewer refuges [70]). Contrasts in terrestriality were defined as same or different using Heldstab et al.’s [73] 3-point categorical classification, with further reference to the primary literature for more nuanced interpretation in cases where both species fall into the same category. In addition, I calculated contrasts in percent time spent on the ground from the compilation in [74]. However, data were available for only 15 of the pairs.

Compared to other primates, brain evolution is unusually well documented within the hominin lineage and covers approximately 4 My of evolutionary history. However, in this case, we are obliged to rely on ECV data as an index of brain mass. I use the ECV volumes and earliest appearance dates from [75], and species body sizes estimated for the same specimens from [76]. Hominin ECV values are reduced by 5.9% (following [77]) to give an improved estimate of actual brain mass to maintain comparability with *Pan* which I use as the baseline contrast. Body mass data for chimpanzees are from [78]; wild chimpanzee brain data are from [79].

### Analysis

The Stephan et al. [63] primate brain dataset yields 31 pairs of tip nodes for which contrasts in brain mass and contrasts in body mass can be calculated. The species and contrasts as used by Deaner & Nunn are listed in the Supplementary Information online S1 Table in S1 File. Deaner & Nunn divided contrast pairs between within-genus sister-species (which will usually have recent divergence dates) and between-genus contrasts (which will have much deeper time depths). This allowed them to have a wide range of divergence dates so as to be able to determine the broad pattern of divergence and subsequent convergence. This is important because, if there is a brain lag effect, we have no idea how long reconvergence on the allometric regression is likely to take.

Deaner & Nunn used the divergence dates given by Purvis [61]. While undoubtedly the best available at the time, these were based largely on anatomical traits and fossil dates. Since then, more accurate molecular genetic phylogenies have become available, and I here use in addition the genetic divergence times provided by Perelman et al. [80]. Although the two divergence estimates correlate significantly (r = 0.732, N = 25, p < 0.0001), the agreement is far from perfect, and on some comparisons (e.g., lemurines versus Old World monkeys) is poor. I compared the Perelman dates with a slightly more recent (but smaller) dataset based on complete mitochondrial genomes [81] and with the median estimates given by the TimeTree website [82], but the differences were marginal (r = 0.908, N = 18, p<<0.0001; r = 0.994, N = 24, p<<0.0001, respectively). I therefore ran the analysis with the original Purvis dates and with the Perelman molecular data.

One central concern is the fact that OLS regression was developed for use with data from dose-response experiments where the values on the X-axis are measured without error because they are determined by the experimenter. This assumption allows the calculation of statistical moments to be simplified because the regression slope can be determined by minimising the residuals against the Y-axis only. However, if the assumptions of the OLS regression model are violated (i.e., when the data are not bivariate normal, or there is significant error variance in the estimates for the X-axis values, or if r^2^<<0.95 due to the presence of grades in the data causing high error variance on both the X- and Y-axes), OLS regression will underestimate the slope, with the underestimate increasing as a negative function of r^2^ [28].

When r^2^ ≥ 0.95, RMA and OLS regression methods converge and give the same answer, and we can use OLS regression. But when r^2^ < 0.95, OLS regression underestimates the slope. In such cases, it is recommended that reduced major axis (RMA), or model 2, regression is used since, by simultaneously minimising the residuals to both axes, the regression is placed up the central axis in the data, rather than across it (see [28] and references therein). To check whether or not Deaner & Nunn’s negative result is an artefact of using OLS regression, I ran both OLS and RMA regressions on the data.

The disadvantage of RMA regression is that there are, as yet, no formal methods for testing whether the slope and intercept parameters differ from the null hypothesis of a = b = 0. However, we can solve this indirectly by a two-step process in which we first ask whether the OLS parameters differ from a = b = 0 (the conventional significance test) and then whether the OLS parameters differ significantly from the RMA parameters, using the standard errors on the OLS intercept and slope to calculate the t-statistic in the usual way in both cases (i.e., in the second step, we treat the RMA slope as the theoretical, or null, hypothesis).

To determine whether the data form natural clusters (statistical grades that have the same slopes but different intercepts), I used *k*-means cluster analysis of residuals from the common RMA regression line. To avoid confounding grades with taxonomic effects, I used as the baseline from which to calculate residuals for the cluster analysis the RMA regression for within-genus contrasts only. This focusses the analysis of residuals on the initial post-divergence phase, thereby avoiding any quadratic effects that might arise later through convergence back onto the allometric regression line.

There are no formal tests for finding *k* (the optimal number of clusters). Goodness of fit inevitably increases with *k* and will, by definition, approach r^2^ = 1.0 when *k* is equal to the number of data points. Convention is to find the value of *k* that maximises fit while minimising the number of clusters, subject to the rule that there should be as few clusters as possible consisting of a single datapoint [28]. To determine the optimal number of clusters, I plot the *F*-statistic as an index of goodness of fit against cluster number. Since this will usually be asymptotic in shape, the point of statistical independence can be identified as the point at which the slope starts to decrease (the point conventionally identified as the ‘break’ in slope in the classic ‘broken stick’ model widely used in ecology). A more sophisticated approach notes that, on any asymptotic curve, the point of inflection (i.e., the point where the magnitude of the change in Y starts to decline relative to the change in X) is defined by the point on the X-axis that corresponds to the point on the Y-axis that is 1/e^th^ down from the asymptote [83]. I use the latter criterion since it is mathematically more precise and easier to calculate; it yields results that are identical to the broken stick method [28].

Having determined whether or not there is a brain lag effect, I ask two further questions: (1) how do the species concerned deal with the additional energetic demands of larger brains and larger bodies and (2) what selection forces drove the deviation from the allometric relationship. Deaner & Nunn tested whether the residuals in the brain/body size relationship predicted social group size as a potential selection pressure for deviating from the allometric regression line. However, primates exploit both increased body mass and increased group size as solutions to high predation risk, and these yield two very different evolutionary equations because the first involves only a conventional energetic demand whereas the second explicitly involves an expensive cognitive demand as well as an energetic demand. The structure of the alternative hypotheses have the form:

(1) Predation risk selects for larger body size [and brain size simply readjusts later] versus
(2) Predation risk selects for larger group size, and group size in turn selects for larger brain size (because of the cognitive demands in coordinating large groups [25,84]).

These are not necessarily mutually exclusive options: some taxa may prefer one strategy over the other (especially if one is less costly) or they may occur sequentially in a lineage if, for example, increases in body size create spare energetic capacity that enables the second. To test these possibilities, I compare contrast residuals to both an index of dietary efficiency (percentage of fruit in the diet) and a well established index of habitat predation risk (degree of terrestriality).

Finally, I ask whether the findings from this analysis explain the diachronic changes that have occurred in the hominin lineage since the hominin/*Pan* split (~7.0 Ma). In this case, all contrasts are between individual hominin species and living chimpanzees (*Pan*). As the time base, I calculated the time elapsed from the divergence of the hominin and chimpanzee lineage at ~7 Ma to the appearance of the earliest member of each subsequent species in the hominin lineage. Dates for the last common ancestor vary between 6–8 Ma, but the difference is of little importance because we use the same reference point for all contrasts: errors in the dating will simply move the distribution of points to the left or right on the X-axis, and only by at most a matter of a million years. This will have not affect whether or not the regression is significant.

Where a directional prediction is being tested, 1-tailed p-values are given and so indicated; all other statistical tests are 2-tailed. Where separate tests are run on different grades, I use Fisher’s meta-analysis for small samples [85] to combine the results: this is a maximum likelihood test that uses 1-tailed p-values to ask how likely it is that the set of individual test results would be as extreme as those observed if there were no underlying trend in the data (with df = twice the number of tests sampled).

### Statement of ethics

An ethics statement was not required for this study since no human or animal subjects or materials were used. All data are from secondary sources.

## Results

The analysis proceeds in three steps. First, we seek to ascertain whether or not there is a brain lag effect, given the availability of more accurate divergence dates and the use of more appropriate statistical methods. There are three possible outcomes: (i) zero correlation if Deaner & Nunn were right and there is no lag effect (i.e., brain and body size always change together in a tight co-evolutionary ratchet); (ii) a significant positive correlation if there is a lag effect in which body size changes first (as predicted by [17]); and (iii) a significant *negative* correlation if there is a lag effect with brain size changing first (in effect, a body size lag). If the answer is (ii) or (iii), we proceed to a second step and ask whether, after the initial deflection, the regression line converges back onto the allometric line (i.e., a simple lag effect) or extends beyond it (i.e., something else is going on). If the second is the case, then, as a third step, we ask (i) how do the species concerned solve the energetic shortfall that is an inevitable consequence of increasing brain size beyond that predicted by brain/body size allometry and (ii) why does this pattern occur rather than taking the form of a simple lag effect.

### Is there a brain lag effect?

I first consider the original Deaner-Nunn analysis with the Purvis divergence dates to check whether an RMA regression changes their results. If the data are bivariate normal and r^2^ > 0.95, then an RMA regression will produce exactly the same best fit equation as an OLS regression. If, on the other hand, there are grades in the data (and r^2^<<0.95 as a result), then the RMA slope will be significantly steeper than the OLS slope. In this case, the RMA regression will always give a truer estimate of the slope. I then ask whether more recent dating of divergences makes a difference.

The original Deaner-Nunn data with the Purvis divergence dates are plotted in the Supplementary Information online Fig. S1 in S1 File. The regression is positive but does not differ significantly from *b* = 0 (b = 0.002 ± 0.004sem, standardised *β* = 0.101, r^2^ = 0.010, F_1,23_ = 0.23, p = 0.316 1-tailed), just as Deaner & Nunn found. However, an RMA regression of these data (Residual = −0.166 + 0.018*Date) yields a significantly steeper slope than the OLS regression (t_23_[0.018 = 0.002 ± 0.005sem]=41.0, p<<<0.0001), with a significantly more negative intercept (t_23_[−0.166 = −0.007 ± 0.051sem]=3.12, p = 0.0024). In other words, the regression model used makes a significant difference. This is usually a signal that a dataset is not homogenous, but consists of a set of grades.

**Fig 1:**
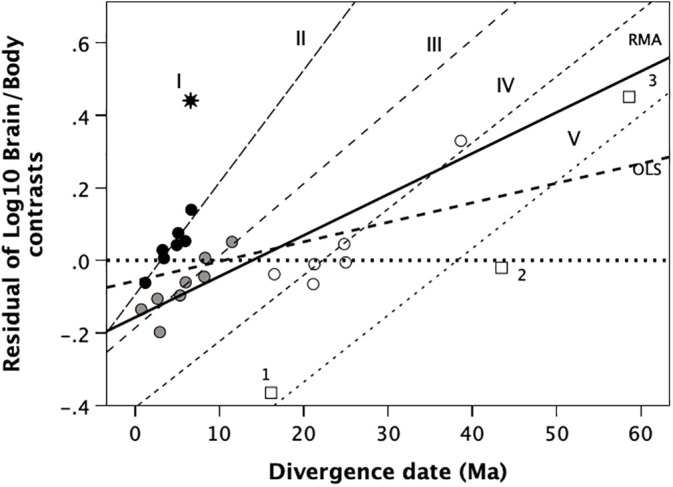
Residuals of the contrasts in log_10_ brain mass regressed on contrasts in log_10_ body mass, plotted against time of divergence for the Deaner-Nunn species pairings (using the Perelman divergence datings). The thin horizontal line is the null hypothesis (b = 0); the heavy dashed line is the OLS regression for the plotted data; the heavy solid line is the RMA regression. A *k-*means cluster analysis of residuals from the within-species RMA regression line indicates that the data form five distinct grades; the contrasts allocated to the grades by the clustering algorithm are indicated by the different symbols (and the Roman numerals by the respective grade OLS regression lines). The symbols differentiate the distinct clusters revealed by the *k*-means cluster analysis. Star: grade 1 (*Homo-Pan*); filled circles: grade 2 (within-genus contrasts); grey circles: grade 3 (within-genus contrasts); unfilled circles: grade 4 (between-genus contrasts); squares: grade 5 (between-family contrasts) (1: *Aloutta/Lagothrix*; 2: *Piliocolobus/Leontopithecus*; 3: *Daubentonia/Avahi*).


Fig 1 plots the residuals for the same contrasts in brain mass regressed on body mass against the updated Perelman divergence times. The solid horizontal line gives the null hypothesis (slope b = 0), and the heavy hatched line is the OLS regression. The OLS regression with the Perelman divergence dates is positive and significant (b = 0.005 ± 0.002sem; standardised *β* = 0.445; r^2^ = 0.198, F_1,23_[*β* = 0.445 > 0]=5.68, p = 0.013 1-tailed). Note that the intercept is negative (body size changes first), though not significantly so (*a* = −0.057 ± 0.045sem; t_23_ = −0.1.26, p = 0.111 1-tailed). The RMA regression through these data (heavy solid line) is:

$$\begin{array}{c} \text{Residual }=\;-\text{0}.\text{1574 }+\;\text{0}.\text{0113}*\text{Date}. \end{array}$$

Its slope is significantly steeper than that of the OLS regression (t_23_[0.0113 = 0.005]=3.15, p = 0.0022 1-tailed), with an intercept that is significantly lower (t_23_[−0.157 = 0.057]=2.23, p = 0.018 1-tailed; and hence also significantly below 0). As with the Purvis datings, an RMA regression yields a significantly steeper slope with a more negative intercept than an OLS regression, again suggesting that there may be grades in the data. The main conclusion, however, is that more accurate dating of divergence times significantly improves the outcome estimates, revealing a lag effect.

To determine whether the data are better described as a series of statistical grades than as a homogenous dataset, I ran a *k*-means cluster analysis on the residuals for Fig 1, using the RMA regression for all the within-genus contrasts as the baseline against which to calculate residuals:


$$\begin{array}{c} \text{Residual }[\text{within}-\text{genus brain}/\text{body}]\;=\;-\text{0}.\text{14106 }+\;\text{0}.\text{017439}*\text{Date} \end{array}$$ [1]

With residuals calculated from eq. [1], a k-means cluster analysis was run for 2 ≤ *k* ≤ 7. All cluster divisions tested provide significant fits to the data (ANOVA, p < 0.001). To find the clustering pattern that maximises fit while minimising the number of clusters, we find the phase transition point in the distribution of goodness of fit as a function of *k* (number of clusters). Online Fig. S2 in S1 File shows that this is at *k* = 5.

Fig 1 plots the five clusters so identified as different symbols (and associated Roman numerals). Since r^2^ ≈ 0.95 for individual grades, OLS regressions are shown in each case. One cluster (the *Homo-Pan* contrast) contains just a single member. Pairwise comparisons between successive grades indicate that, as a set, the slope parameters do not differ significantly across the four grades (Fisher’s meta-analysis: p = 0.177), but the intercept parameters do (p = 0.0008) (Table 1). Notice that all but two of the datapoints in the two leftmost grades (labelled II and III) are congeneric species; and all but one of the datapoints in the two rightmost grades (labelled IV and V) are comparisons between species that belong to different genera, families or suborders.

**Table 1:** Pairwise comparisons of slope and intercept coefficients of the regression equations for the grades in Fig 1.

Comparison	slopes (±se)	intercepts (±se)	df
II vs III	0.024 ± 0.008 vs 0.017 ± 0.005 1.077, p = 0.151	−0.060 ± 0.038 vs −0.177 ± 0.036 t=3.162, p=0.004	13
III vs IV	0.017 ± 0.005 vs 0.017 ± 0.005 t=2.186, p=0.025	−0.177 ± 0.036 vs −0.365 ± 0.133 t=0.000, p=0.500	12
IV vs V	0.017 ± 0.005 vs 0.019 ± 0.008 t=0.308, p=0.500	−0.365 ± 0.133 vs −0.681 ± 0.309 t=1.430, p=0.098	7
Fisher’s test*:			
χ^2^ (df=6)	8.948	23.066	
p	0.177	0.0008	

* Fisher’s meta-analysis for small samples: χ^2^ = Σln(p/2) (df = twice the number of tests) testing for a set of one-tailed p-values that, as a set, are significantly more extreme than p = 0.05. All p-values are given as 1-tailed.

Grades II-V have individually positive and significant or near-significant regression slopes. The mean correlation across the four grades is r = 0.933 (taking grade into account, overall r^2^ = 0.892, mean standardised β = 0.936: Fisher’s procedure for combining independent tests: χ^2^ = 19.2, df = 2*4 = 8, p = 0.013, indicating a consistent underlying positive trend). As is invariably the case when grades are present, failing to take them into account results in an underestimate of the true regression slope, as well as the goodness of fit [25,28]. The OLS regression for the full dataset yields a standardised slope of β = 0.445; taking grades into account yields a value (β = 0.936) that is significantly steeper (one sample t-test: t_3_[β = 0.445]=31.58, p<<0.0001).

Three points may be noted about the distributions in Fig 1.

**Fig 2:**
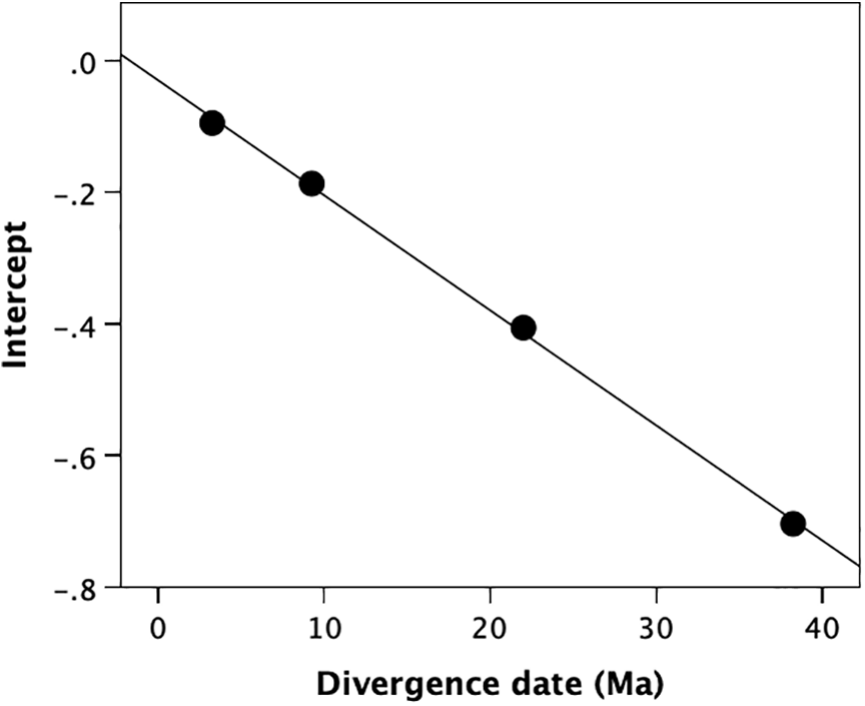
Intercept values for the regression equations for the four grades in Fig 1, plotted against mean Perelman divergence dates (indexed by the date at which the grade’s regression line crosses the origin line at residual = 0). The mean divergence date is calculated as the date at which the grade OLS regression crosses the residual = 0 line.

**Fig 3:**
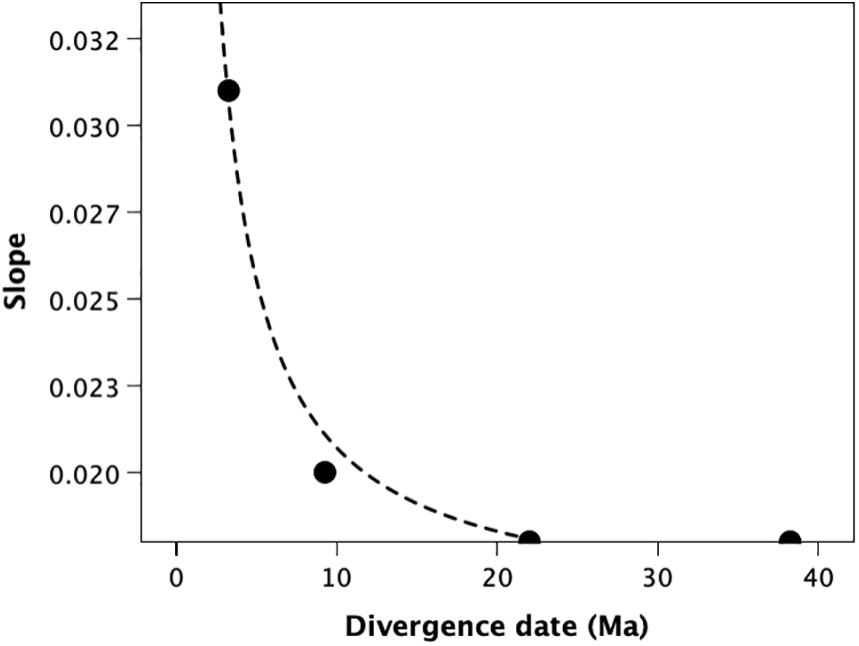
Slope values for the regression equations for the four grades in Fig 1, plotted against mean Perelman divergence dates (indexed by the date at which the grade’s regression line crosses the origin line at residual = 0; calculated as in Fig 2).


First, in each grade, the most recent contrasts have a negative residual, while the older ones have positive values. For the two mainly within-genus grades (II and III), the distribution of datapoints in grade II is displaced upwards compared to the earlier grade III: in grade II, only 1/7 datapoints falls below the zero line (where brain size is what would be expected for body size), but 6/8 do so in grade III. In other words, the intercepts become increasingly more negative as the divergence time of the grade gets deeper (i.e., more rightwards on the graph) (Fig 2; r^2^ = 0.999, standardised β = −1.0, t_2_ = −47.9, p = 0.0004 2-tailed). The regression equation for the data in Fig 2 is significantly different from the null hypothesis of no correlation (i.e., slope b[H_0_]=0) (r^2^ = 0.999, standardised β = −1.0, t_2_ = −47.9, p = 0.0004 2-tailed). Notice how tightly the data points cluster along the regression line. In contrast, the slope parameters are significantly steeper in more recently diverged lineages (Fig 3: r^2^ = 0.983, standardised β = −0.991, t_2_ = −16.7, p = 0.002 2-tailed). The best fit regression is in fact an inverse equation:

$$\begin{array}{c} \text{Slope }=\;\text{0}.\text{0159 }+\;\text{0}.\text{0480}*{\text{Date}}^{-\text{1}} \end{array}$$

(r^2^ = 0.983, t_2_ = 10.67, p = 0.002 2-tailed). This suggests that younger divergences favour investing disproportionately in larger brains, whereas older ones have preferred to increase body size.

The second point to note is that the time lapse to converge on the allometric line is 2.0 million years for grade II and 8.6 million years for grade III. Notice, also, the high value for the *Homo/Pan* contrast. (Deaner & Nunn chose to discount this from their analysis, probably because it was so divergent.) Its position suggests that it might represent the end-point of a separate grade whose starting point is much higher than that for grade II (see below).

The third point is that, although the brain/body ratios for grades IV and V do seem to stabilise back on the regression, those for the younger grades II and III clearly do not. Instead, they continue to diverge past the allometric line in favour of increasingly large brains. The contrasts for grades IV and V are phylogenetically much deeper. All but two of the contrasts in grade IV are strepsirrhines (the other two being New World monkeys and a great ape pair, both with unusually deep divergence times). Since the deep strepsirrhine contrasts cluster around the zero line, they appear to have converged on the allometric regression. It seems only to be anthropoids that extend beyond the allometric regression line into positive brain size territory (grades I-III). This suggests that the anthropoids exhibit a phase shift in favour of larger brains in their response to the environmental drivers that influence brain and body size. More importantly, within the anthropoids, there is a further phase shift in favour of large brains among some lineages (mainly cercopithecines and apes, but also including some platyrrhine lineages).

This is in line with evidence that the strepsirrhines do not exhibit a strong social brain relationship [19,24,25,53].

### How the energetic constraint was resolved

A significant deviation from the common allometric regression line necessarily creates an energetic demand to fuel the additional body or brain growth. Of these, brain growth is the more important because brain tissue is ~ 20 times more energetically costly than somatic tissue (the expensive tissue hypothesis [86–89]). One solution to this is to switch to a more nutrient-rich diet. Primates broadly divide into folivores and frugivores, with frugivory usually considered the richer, more energy-accessible diet. In contrast, a folivorous diet requires fermentation by specialised bacteria to enable the animal’s digestive system to access the nutrients contained within the cell walls of leaves [90]. This imposes severe constraints on both the volume of nutrients that can be processed and the efficiency with which nutrients can be extracted: folivores are forced to stop feeding once the stomach is full in order to allow digestion via foregut fermentation [90], an activity that is incompatible with all other activities [36].

**Fig 4:**
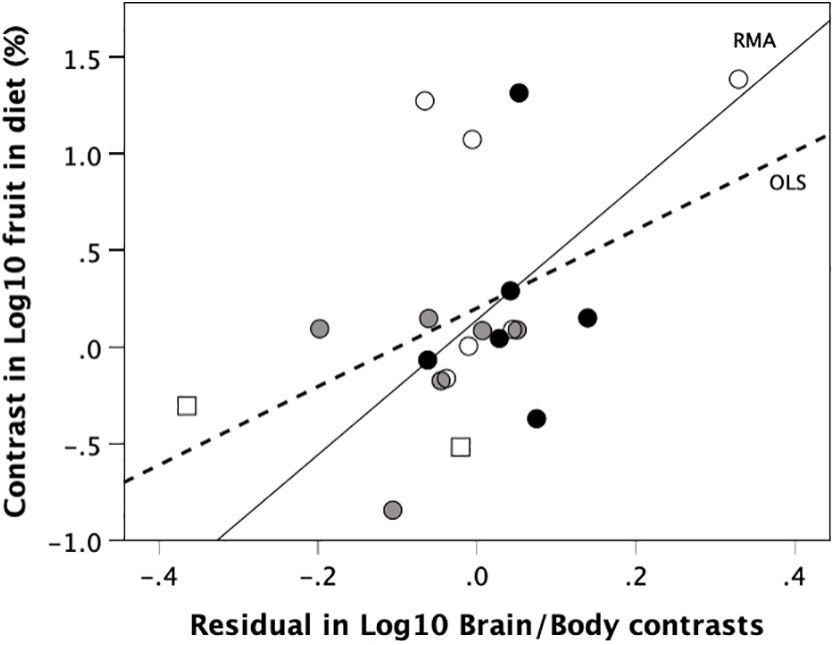
Contrast in percent fruit in diet plotted against residual of contrast in log_10_ brain volume from contrast in log_10_ body mass. Dashed line (OLS): ordinary least squares regression; solid line (RMA): reduced major axis regression. Symbols as for Fig 1.

To test whether the switch from investing in body size to investing in brain size is associated with a change in diet, Fig 4 plots contrasts in percent of fruit in the diet against the residual of contrasts in brain size regressed on contrasts in body size. The OLS regression is positive and significant (r = 0.415; F_1,22_ = 4.59, p = 0.022 1-tailed): species with larger brains for body size are more likely to be frugivorous. I compared the percentage of fruit in the diet separately for contrasts where brain size residuals were positive versus negative (online Fig. S3 in S1 File). Overall, the difference is not significant (t_22_ = 1.06, p = 0.151 1-tailed). However, most strepsirrhines are insectivorous and belong to a smaller-brained sub-order than haplorrhines. Excluding strepsirrhines yields a significant difference within the anthropoids (t_16_ = 2.05, p = 0.029 1-tailed). This suggests that when the anthropoids opted for an increase in brain size, a change in diet to a more nutrient-rich regime was required, but this was not the case when they opted for an increase in body size – presumably because the energy demand was much lower in the latter case. It seems that a dietary adjustment of this kind was not necessary for the strepsirrhines, perhaps because their much smaller brains require less energy and because they do not deviate so far from the allometric regression (in effect, after increasing body size, they simply converge back onto the allometric regression line: Fig 1).

### Was predation risk the selection pressure involved?

**Fig 5:**
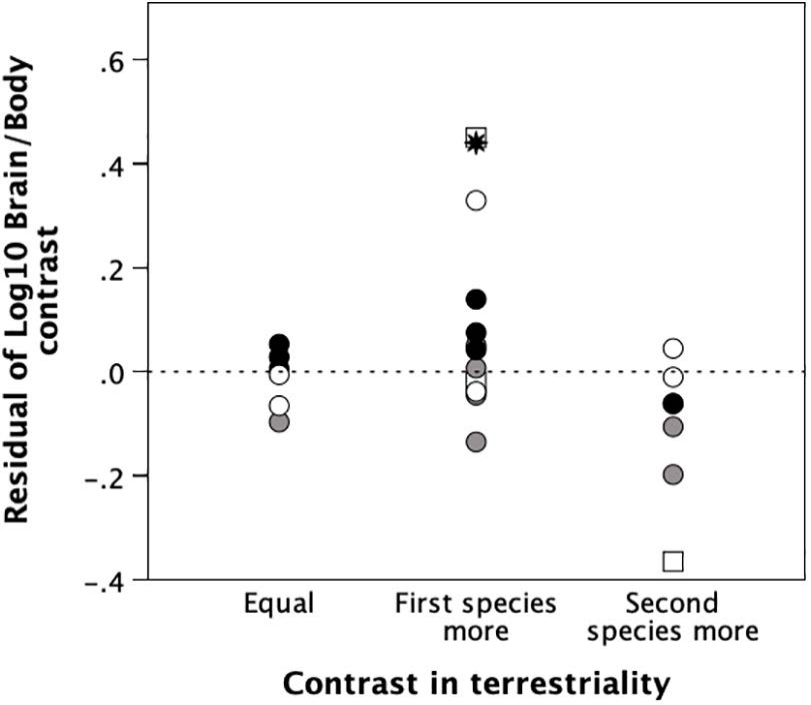
Residuals in the contrasts in log_10_ brain size regressed on log_10_ body size as a function of Helbstad’s index of relative degree of terrestriality. Dashed horizontal line indicates residual = 0 (brain size lies on the allometric line). Symbols as for Fig 1.

**Fig 6:**
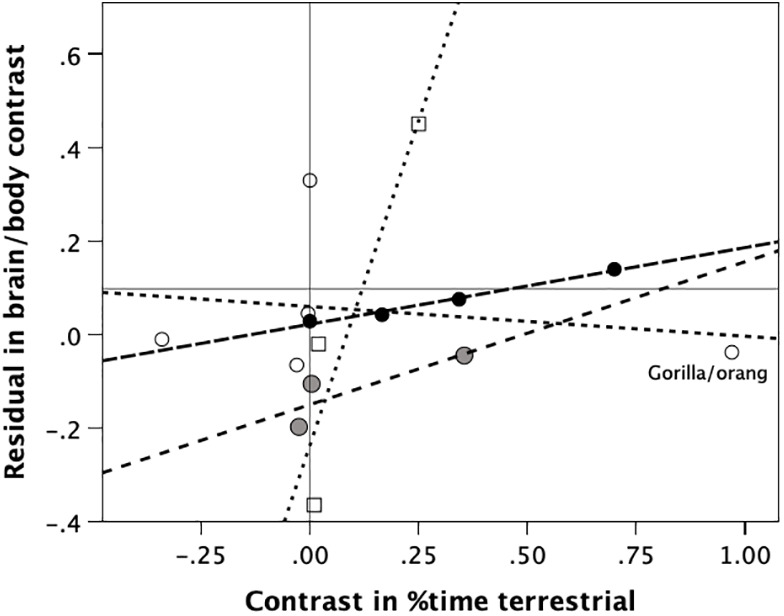
Residuals in the contrasts in log_10_ brain size regressed on log_10_ body size as a function of percent time spent terrestrial. Symbols and OLS regressions for individual grades as for Fig 1.

To test the hypothesis that the underlying driver for the changes in brain and body size is predation risk (indexed by the species’ degree of terrestriality), I plotted contrasts in brain:body mass as a function of contrasts in the terrestriality index (Fig 5). For contrasts where there is no difference in terrestriality, residual brain size values cluster around 0, but residual brain size is significantly different when one of the pair is more terrestrial (t_21_ = 2.61, p = 0.016). This suggests that predation risk is likely to have played a role in triggering changes in both brain and body size. There are only limited quantitative data available on terrestriality (Fig 6), but what there is suggests that the percent of time spent on the ground correlates positively with residual brain:body contrasts in three of the four grades (albeit with only grade II individually significant: p = 0.0035). Nonetheless, a Fisher meta-analysis indicates that, overall, the data are significantly more positive than would be expected if there was no underlying trend (χ^2^ = 19.79, p = 0.0112).

**Fig 7:**
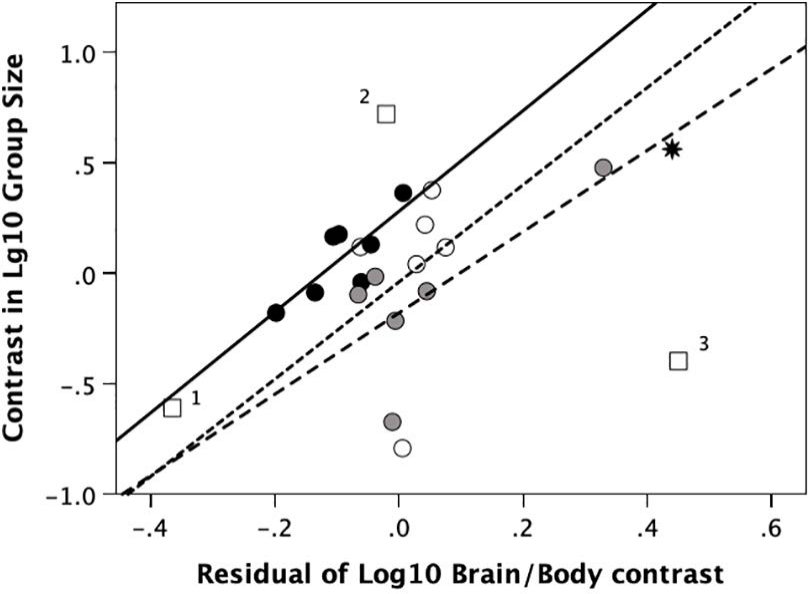
Contrasts in log_10_ mean group size plotted against residual of contrast in log_10_ brain mass regressed on contrast in log_10_ body mass. Symbols as for Fig 1. Regression lines are for grades II (solid line), III (long dashed line) and IV (short dashed line), as defined in Fig 1.

Deaner & Nunn [17] tested for a relationship between social group size and residual brain size, but found none. However, using more up-to-date data on species’ mean group sizes from [68], there is in fact a highly significant positive overall correlation (Fig 7: r = 0.612, p = 0.005 2-tailed). All three grades where N > 3 exhibit positive slopes (r = 0.806, p = 0.015; r = 0.410, p = 0.312; r = 0.724, p = 0.052; Fisher’s procedure: χ^2^ = 16.64, df = 6, p = 0.011, indicating a consistent positive trend across the three subsamples).

Note that two of the squares in Fig 7 lie well off-diagonal, which is usually indicative of a grade shift. The higher one (datapoint 2) represents a contrast between *Piliocolobus* (a semi-terrestrial Old World monkey) and *Leontopithecus* (an arboreal New World monkey) that parallels a shift in sociality between a highly social grade (genera include gibbons, *Papio*, *Macaca*, terrestrial guenons, atelids, callitrichids) and a much less social grade (Asian leaf monkeys, arboreal guenons, *Colobus*, cebids, tamarins, *Saimiri*) – essentially the same genera that fall on the more and less social grades of the social brain data (see [25]).

The lower datapoint (3) is a contrast between two distantly related strepirrhines (*Daubentonia* and *Avahi*). For present purposes, and to be conservative, I followed Deaner & Nunn [17] and others in assigning a group size of N = 1 to *Daubentonia* on the grounds that it usually forages alone. However, there are strong grounds for believing that this species actually lives in dispersed communities as large as N ≈ 8 [25]. If their correct group size is anywhere close to 8, it would place this contrast very close to the star (*Homo-Pan* contrast), yielding a slight improvement in overall linear fit (r^2^ = 0.622, p = 0.004). The important point here, however, is that even if this datapoint is subject to a great deal of measurement error the overall relationship remains significant.

**Fig 8:**
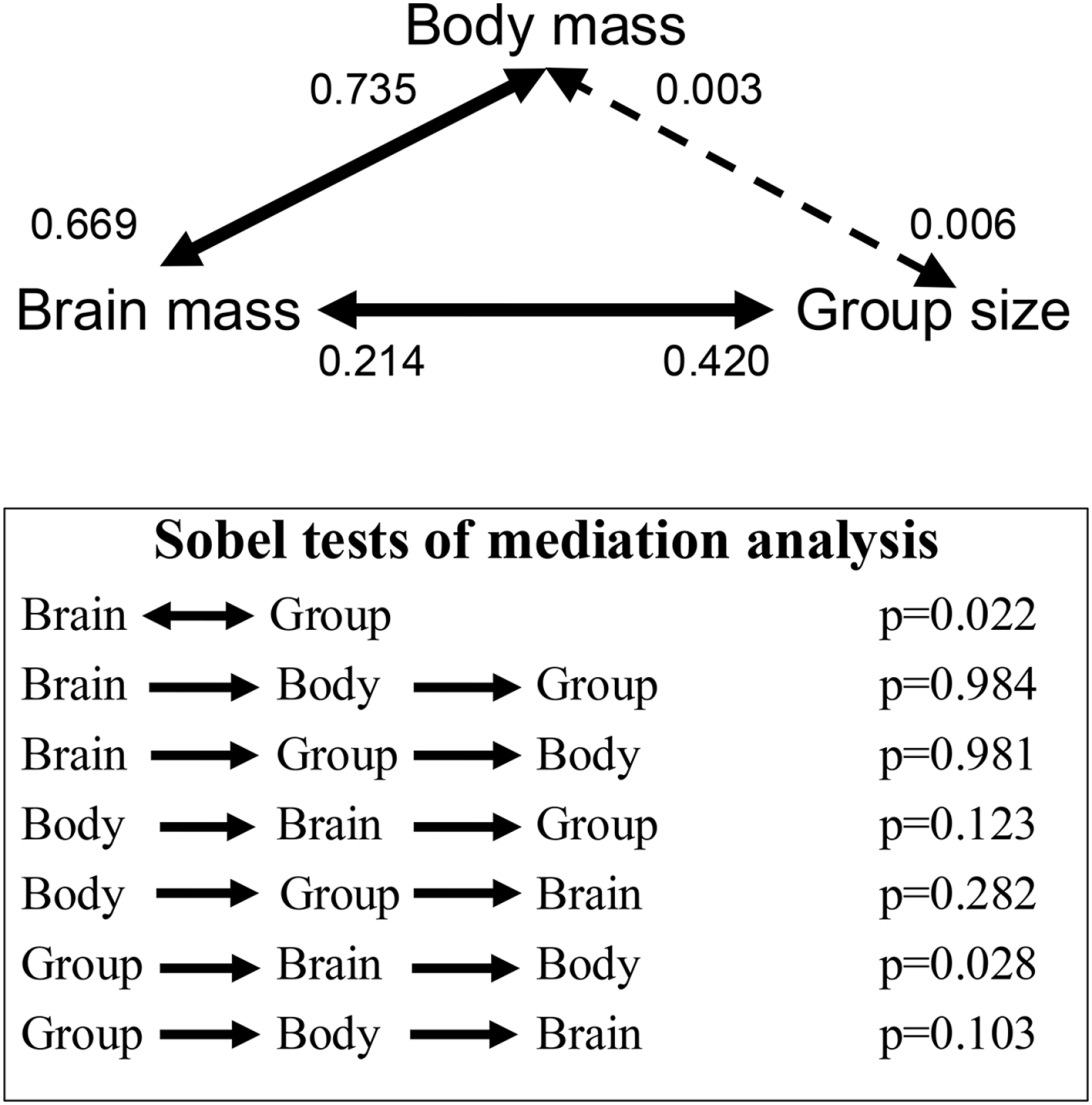
Path analysis of the functional relationships between contrasts in log_10_ brain mass, contrasts in log_10_ body mass and contrasts in log_10_ social group size, based on multiple regressions for each in turn as the dependent variable. The numbers adjacent to the arrow heads are the standardised slopes (ßs) in each direction (where the adjacent arrowhead specifies the dependent variable). Solid lines indicate significant predictors; dotted lines indicate non-significant predictors. Sobel tests of mediation analyses are given for all six possible causal (i.e., directional) sequences, and for bivariate regression between contrasts in brain size and contrasts in group size.

Deaner & Nunn [17] implicitly assumed a causal relationship in which changes in body size drive changes in brain size, with changes in group size being presumably at best a default by-product of changes in brain size. A path analysis of the relationships between these three variables (based on significant standardised slopes in multiple regressions with each variable in turn as the dependent) yields a best fit model in which brain size is correlated independently with body size and group size, with no relationship between body size and group size (Fig 8). Importantly, mediation analysis for the six possible three-way causal pathways indicates that the only significant pathway is group size predicting (driving) brain size, and brain size predicting body size (Sobel test: p = 0.028); none of the other alternative pathways are significant (p ≥ 0.103). In other words, group size selects for an increase in brain size, and a larger brain then selects for an increase in body size in order to exploit Kleiber’s Law to reduce the energetic costs. This would seem to suggest that, aside from the exceptions noted above, body size is only rarely exploited as a first line defence against predators, at least by anthropoid primates. Instead, most species opt directly for group-living as the solution, with body size being one way in which the energetic demands of brain growth can be offset.

### Hominins as a test case

**Fig 9:**
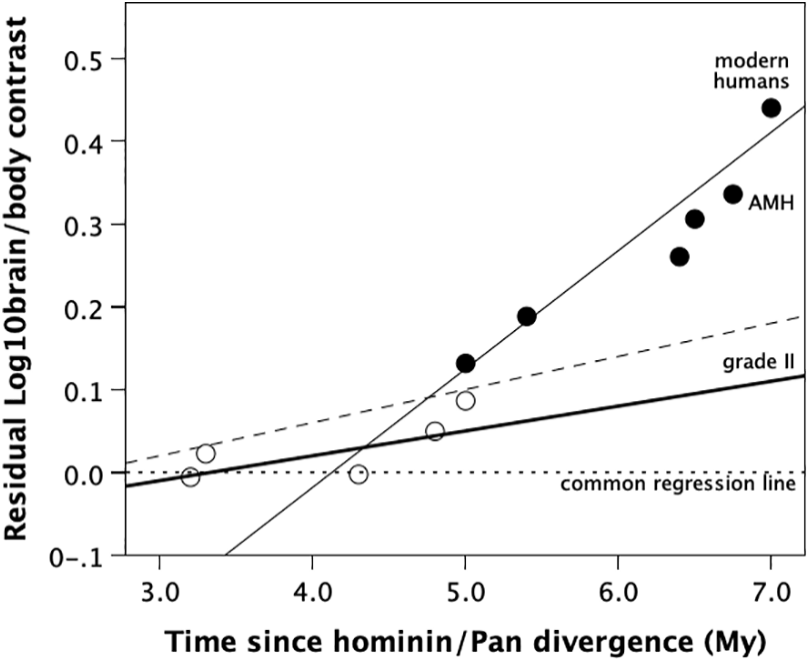
Residual of hominin/*Pan* contrast in Log_10_ brain volume regressed on contrast in Log_10_ body mass for individual fossil hominin species, plotted against time since *Homo/Pan* divergence at ~7 Ma. The modern human datapoint is the *Homo/Pan* contrast from Fig 1; the increase in residual brain size compared to fossil *Homo sapiens* (AMH) is due to a marked reduction in human body size in modern populations. Dating is to midpoint of species time span. For this analysis, brain volume is based on ECV, and has been corrected by ~6% to adjust for the fact that ECV overestimates actual brain mass; in most cases, body mass is estimated from long bone diameter (a reliable index of the body mass an animal has to carry during locomotion). Unfilled symbols: australopithecines; filled symbols: *Homo*. The horizontal dotted line is the common primate regression line. Thick solid line: grade II regression line, with the dashed line giving the upper 95% CI around this regression (from Fig. 1). Thin solid line: OLS regression set to data values >4.0 My. AMH: fossil anatomically modern humans, dating to ~100 ka.

The hominin lineage is one of the few cases where the fossil record allows us to trace the evolutionary pathway in considerable detail. *Homo* derives from an ape-like australopithecine root with a body size indistinguishable from that of modern *Pan* [91]. Fig 9 plots the change over time in the brain:body mass residual from the primate RMA regression line (in this case, for want of any more suitable alternative, using corrected ECV to estimate brain mass). The australopithecines fit comfortably on the grade II regression line (and well within its 95% CIs), but the appearance of *Homo* around 2.5 Ma marks a phase shift with a steep upturn in brain size giving rise to a dramatic increase in the brain:body mass residual. The best fit OLS regression has an inverse form (r^2^ = 0.724, standardised β = 0.851, t_9_ = −4.857, p = 0.0009 2-tailed), although excluding the two earliest australopithecine datapoints gives a significant linear fit (thin solid line: r^2^ = 0.978, p < 0.0001).

There is some suggestion that the australopithecines initially undertook a move towards relatively larger body size (residuals below the grade II primate regression line), and then, once the more nomadic early *Homo* began to occupy increasingly open (high risk) environments from around 2.5 Ma, hominins underwent an explosive increase in brain size without corresponding changes in body size that catapulted their brain:body size ratio far beyond the allometric regression. This correlates closely with a dietary shift towards a more nutrient-rich meat-based diet from ~2.0 Ma, initially in the form of scavenging in early *Homo* but later in the form of active hunting among archaic and modern humans from ~0.6 Ma.

## Discussion

Although Deaner & Nunn [17] found no evidence for a brain lag effect (changes in brain size lagging behind changes in body size) in primates, a statistically more sophisticated analysis of their original data, with and without updated divergence times, suggests that there is in fact a significant positive correlation as predicted by the brain lag hypothesis. More importantly, an appreciation that the brain data exhibit grade effects rather than being a single homogenous dataset [21,25,26] yields a much stronger relationship. Changes in body size do seem to precede changes in brain size.

The subsequent increase in brain size does not, however, involve a simple convergence back onto the equilibrium brain/body ratio in the way the original brain lag hypothesis assumed. Instead, at least in anthropoid primates, it involves continued increases in brain size beyond the main allometric regression to a point where brain size is consistently and stably larger than expected for body size. This result is in line with earlier findings that grades within the social brain dataset involve a stepwise emphasis on neurally expensive socio-cognitive skills [25,84]. This is also in line with recent evidence that the principal changes in primate brain organisation mainly involve the prefrontal and temporo-parietal regions and the DMN [92,93], all of which are specifically associated with social cognition. It is important to appreciate that the grades identified in the present study are not taxonomic in origin in the way that phylogenetic analyses assume (see [25]). Although there is a modest taxonomic component, they mainly seem to represent attempts by individual species, sometimes genera, to manage the specific environmental challenges in the habitats they happen to gain access to.

These results would seem to be in line with Lande’s [2] finding that the variance in the primate allometric relationship increases with body size, suggesting that the two become progressively decoupled as body size increases [19,33,34]. This might explain how brain size escapes from the energetic constraint imposed by body size in species with large body sizes (mainly those in grades I and II in Fig 1). What may have been critical in facilitating this was a shift to more nutrient-rich (i.e., frugivorous) diets, itself dependent on being able to cope with longer day journeys since edible fruits are more sparsely distributed than leaves [36,92]. One important implication of this decoupling of brain and body size, especially among the larger-bodied species, is that the use of relative brain size (the Encephalisation Quotient) may be seriously misleading [28,93–96]. It is difficult to know why the concept continues to persist in the literature.

The original brain lag hypothesis assumed that the common allometric regression acted as an attractor to which the brain:body size ratio inevitably converges if deflected. Since there is a strong correlation between group size and brain size [23–26], this could imply that group size simply increases in response to the availability of additional brain tissue. The present results indicate that this is unlikely. Fig 6 indicates that the causal logic is in fact the reverse: brain size increases under selection from the need to increase group size in response to novel ecological challenges, and the demand for an increase in brain size then selects for an increase in body size to provide the required spare nutrient capacity (Fig 8).

These trends in the brain/body relationship seem to be mainly a response to heightened predation risk as species occupy more risky habitats. This is especially clear in the hominin case: the regression line in Fig 9 takes off at the 2.5 My mark when early *Homo* adopted a more nomadic lifestyle (associated with fully developed bipedal striding) and occupied more open habitats beyond the woodland and riverine habitats favoured by the semi-arboreal chimpanzees and australopithecines. Although primates exploit both large body size and large group size as anti-predator strategies [36–40], they face a particular problem in the latter respect: the bonded nature of primate social groups [21,25,84] means that stable changes in group size are difficult to effect because the personalised relationships that underpin these groups involve complex neural pathways in the brain [58,97,98].

This suggests that increases in body size may be easier to effect, and hence invariably appear first; once in place, however, they may provide an energetic platform that generates sufficient spare capacity derived from savings of scale via Kleiber’s Law to allow species to invest disproportionately in larger brains should they need to. When combined with switches to a more nutrient-dense diet, this allows brain size to break through the brain/body size equilibrium threshold of the allometric regression, permitting much larger groups to form. This trajectory is well illustrated by the historical changes in relative brain size in fossil hominins (Fig 9).

Two important features of this process are worth emphasising by way of conclusion. First, the way the grades in Fig 1 and online Fig. S3 in S1 File are staggered suggests a ratcheted effect in which the earliest transitions largely emphasised body size over brain size, whereas later transitions were able to build on changes already in place to catapult species onto a higher cognitive plane in which group size replaces body size as the main anti-predator strategy. Second, the switch from large body size to large brain size appears to take place over a much longer time period than we might expect for most anatomical changes. In the two within-genus grades (II and III), this switch is distributed over periods of 2 and 8 My, respectively (Fig 1). This suggests that, as species seek to extend the range of ecological niches they can occupy into ever more predator-risky habitats, there was a form of directed evolution in response to specific selection pressures which has to act against considerable evolutionary inertia (represented by the costs involved in both the higher energetic demands of larger brains and the need for considerable neural restructuring).

The present results reinforce recent findings [19–21] that there are shifts in the allometric relationships that occur within the mammals (as Jerison [1] originally pointed out). Across mammals in general, Smaers et al. [20] concluded that these shifts do not always involve cognitive (i.e., brain size) responses to environmental conditions. Different mammalian lineages exhibit different slope parameters in the steepness of the brain/body correlation that are, in fact, best predicted by the likelihood of having bonded social groups, as was previously demonstrated [21]. Smaers et al. [20] were unable to offer any convincing explanation for these grade shifts, but suggested (on the basis of differences in terrestriality) that it may be related to locomotory style (albeit without offering any reasons why locomotory style might be so neurologically demanding) (see also [99]). Perhaps lack of familiarity with mammal natural history caused them to overlook the fact that terrestriality is highly correlated with the occupation of habitats that lack easy access to refuges, that in primates high predator density and lack of refuges correlates highly with social group size, and that living in large social groups is cognitively extremely demanding [25,84]. As several recent analyses have shown, increases in brain size within the primate lineage were mainly associated with changes in brain units specifically associated with sociality and social skills (the DMN and the prefrontal and temporo-parietal cortices [97,98]), and more generally with increasing integration across cortical units (itself a function of expansion in the DMN) [100–101].

At root, this reflects the fact that different mammalian lineages have adopted radically different kinds of anti-predator strategies. These have included opting for large body size (the great apes, elephantids, the cetaceans, some ungulates), opting for large social groups (cercopithecine primates, some great apes, delphinids), exploiting locomotor advantages (many antelope, patas monkeys, gibbons) and crypsis (the smaller artiodactyls, the nocturnal strepsirrhines) [36,102]. These differ in terms of their efficiency as anti-predator strategies and the costs required to evolve and maintain them. Of the latter, the cognitive costs of social bonding are by far the most expensive energetically [25,83]. Evolution is always a trade off between the benefits that accrue from a strategy and the costs of implementing the strategy. Different taxonomic groups may choose to optimise their solutions in different ways.

The dramatic expansion in the absolute and relative size of the human brain, especially late on in human evolution [98], has attracted considerable attention, although most studies have struggled to find any convincing ecological explanation. The answer would seem to lie in fact that the phase shift mainly involved expansion of brain regions associated with social cognition (the DMN and the prefrontal and temporo-parietal cortices) [96–98] and the fact that large social groups are extremely difficult to maintain as coherent units [83]. Ecology certainly lies at the root of the need for large social groups (Figs 5 and 6), but it is the psychological problem of maintaining social coherence that has driven the need for a large brain (Fig 8).

In sum, contrary to Deaner & Nunn’s [17] claim that there is no brain lag effect, there does in fact seem to be strong evidence for the hypothesis. However, at least in primates, this is not due simply to brain size “catching up” with body size. Instead, it involved an initial increase in body size and then what seems to have been a progressively greater increase in brain size, such that the brain not merely catches up with body size (as the brain lag hypothesis predicts) but actually extends well beyond the allometric line, placing some taxa onto a new higher cognitive grade. This is a rather different picture to the classic brain lag hypothesis. Because the cognitive demands of group-living are very substantial, this switch to exploiting the benefits of group size is only possible if brain size can increase sufficiently to support the cognitive mechanisms involved [25,83]. The outcome is an explanation that makes more biological sense and offers a more comprehensive explanation not only for the general macro-evolutionary patterns but also for many of the micro-evolutionary patterns within them that are often ignored in comparative analyses.

## Supporting information

S1 File S1 Table. Contrasts in brain and body mass for the sample tip taxa. S2 Table. Contrasts for hominin taxa. S1 Figure. Brain/body contrasts versus Purvis divergence date. S2 Figure. Optimal number of clusters. S3 Figure. Contrasts in diet in relation to brain/body mass ratio.
(DOCX)
