# Supplementary material for: Evolutionary lags in the primate brain size/body size relationship revisited
Source: PLoS One. 2026 Jul 1;21(7):e0351073. doi: 10.1371/journal.pone.0351073 (PMC13322519; doi:10.1371/journal.pone.0351073)
Supplement: S1 File — Contrasts in brain and body mass for the sample tip taxa. S2 Table. Contrasts for hominin taxa. S1 Figure. Brain/body contrasts versus Purvis divergence date. S2 Figure. Optimal number of clusters. S3 Figure. Contrasts in diet in relation to brain/body mass ratio. (DOCX) [file pone.0351073.s001.docx]

**Significance of Evolutionary Lags in the Primate**

**Brain Size/Body Size Relationship**

**Robin Dunbar**

***Supplementary Information***

**S1 Table. Contrasts in brain and body mass for the sample tip taxa**

Genus Contrast* LCA (Ma†) Contrasts in: Terrestrial

Purvis Perelman Brain mass Body mass Diet Lg_10_Group size index contrast

------------------------------------------------------------------------------------------------------------------------------------------------------------------------------

**Within-genus contrasts^§^:**

*Hylobates H. lar/H. agilis* 0.5 3.26 0.041 0.003 0.044 0.04 0

*Ateles A. fusciceps/A. geoffroyi* 0.7 3.42 0.024 0.012 -0.79 2

*Callithrix C. jacchus/C (Cebuella) pygmaea* 3.9 5.96 0.277 0.347 1.314 0.38 2

*Colobus C. polykomos/C. guereza* 0.1 2.65 0.053 0.241 -0.844 0.17 0

*Papio P. anubis/ P. ursinus* 0.6 1.21 0.049 0.163 -0.068 0.12 0

*Macaca M. mulatta/M. fascicularis* 1.6 5.12 0.153 0.109 -0.371 0.12 1

*Cercopithecus C. lhoesti/C. mona* 2.1 8.22 0.134 0.274 -0.175 0.13 1

*Cercopithecus C. mitis/C. ascanius* 3.4 2.94 -0.039 0.241 0.094 -0.18 0

*Cercopithecus C. (Erythrocebus) patas/*

*C. (Chlorocebus) aethiops* 3.5 4.95 0.228 0.285 0.290 0.22 1

*Hylobatids Symphalangus syndactylus/H. lar* 6.3 0.146 0.184 -0.193 -0.07 1

*Cebus C. capuchinus/C. (Sapajus) apella* 17.9 6.00 0.011 0.099 0.147 -0.04 0

*Saguinus S. oedipus/S. midas* 5.34 -0.019 0.109 0.18 2

*Saimiri S. oerstedi/S. sciureus* 0.73 0.060 0.300 -0.09 1

*Cercocebus C. galeritus/C. torquatus* -0.015 0.049 -0.134 -0.17 1

*Presbytis P. cristata/P. obscura* 0.054 0.233 0.31 0

*Galaginae G. (Otolemur) crassicaudatus/*

*G. (Galagoides) demidorph* 1.9 0.463 0.879 0.420 -0.07 (0)

*Lemur L. catta/L. (Eulemur) mongoz* 14.0 21.28 0.036 0.058 0.004 -0.67 0

*Tarsier T. banacus/T. syrichta* -0.125 0.093 0.00 (0)

**Between-genus contrasts:**

*Homo-Pan H. sapiens/Pan* spp. 7.0 6.60 0.531 0.130 0.56 1

*Gorilla/Pongo G. gorilla/P. pygmaeus* 14.5 16.52 0.278 0.297 -0.163 -0.02 1

*Macaca-Allenopithecus M. nemestrina/A. nigroviridis* 9.6 11.50 0.185 0.201 0.088 1

*Mandrillus/Lophocebus M. sphinx/L. albigena* 6.7 6.67 0.278 0.208 0.150 1

*Semnopithecus/Nasalis S. entellus/N. larvatus* 6.3 0.070 0.085 0.084 0.36 1

*Piliocolobus/Leontopithecus P. badius/L. rosalia* 39.0 43.47 0.804 1.325 -0.518 0.72 1

*Alouatta/Lagothrix A. seniculus/L. lagothricha.* 13.7 16.13 -0.270 0.137 -0.305 -0.61 0

*Aotus/Callicebus A. trivirgata/C. moloch.* 21.3 24.82 0.095 0.064 0.090 -0.08 0

Lorisidae *Nycticebus coucang/*

*Loris tardigradus* 12.0 21.14 0.183 0.387 1.273 -0.01 2

Cheirogaleidae *Cheirogaleus major/*

*Microcebus murinus* 10.2 24.99 0.597 0.964 1.073 -0.22 2

Lemuridae *Daubentonia/Avahi* 14.0 58.61 0.635 0.283 -0.40 1

Lemuridae *Varecia/Lepilemur* 20.0 38.64 0.592 0.410 1.385 0.48 1

Indridae *Indri indri/ Propithecus verrauxi* 0.156 0.293 -0.311 -0.17 1

Sources: Deaner & Nunn (1999); diet: Powell et al. (2017) and Dunbar & Shultz (2024); terrestriality: Helbstad et al. (2016); divergence dates: Purvis (1995) and Perelman et al. (2011).

* Contrasts are as specified by Deaner & Nunn (1999)

† LCA: last common ancestor; Ma: million years ago

§ Species considered to be members of the same genus (or very closely allied genera) at the time of the Deaner & Nunn study are retained as within-genus contrasts here

**S2 Table. Contrasts for hominin taxa**

Species LCA to *Pan* Contrasts in

(Ma)* brain mass† body mass

--------------------------------------------------------------------------------------------------------

*Australopithecus aethiopicus* 4.3 0.0416 0.0110

*Australopithecus afarensis* 3.2 0.0586 0.0440

*Australopithecus africanus* 3.3 0.0869 0.0440

*Paranthropus boisei* 4.8 0.1170 0.0490

*Paranthropus robustus* 5.0 0.1539 0.0490

*Homo ergaster* 5.0 0.3180 0.2427

*Homo erectus* 5.4 0.4059 0.2933

*Homo heidelbergensis* 6.4 0.5102 0.3457

*Homo neanderthalensis* 6.5 0.5601 0.3528

*H. sapiens* (fossil) 6.8 0.5770 0.3316

--------------------------------------------------------------------------------------------------------

Sources: dates and brain mass: De Miguel & Henneberg (2001); body mass: Will et al. (2017);

*Pan* brain mass from Cofran (2018); *Pan* body mass from Pusey et al. (2005)

*** Million years ago

† ECVs, corrected following Aiello & Dunbar (1993)

*
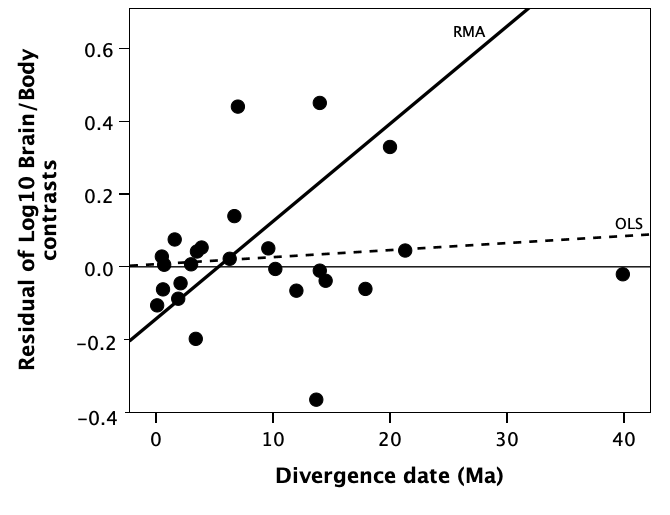
*

**S1 Figure.** **Brain/body contrasts versus Purvis divergence date.** Analysis of contrasts data plotted against divergence time, using the Purvis et al. (1995) phylogeny and estimates of divergence date. The thin horizontal line demarcates a slope of ***b***=0. The thick dashed line is the OLS regression through the data; the thick solid line the RMA regression line.

Figure S1 plots the residuals of the contrast in brain/body mass from the overall regression against the divergence dates from Purvis (1995), as used in the Deaner & Nunn (1995) analysis. The OLS regression through the data give a best-fit regression equation of:

Residual = 0.007 + 0.002*Date

(r^2^=0.010; t_23_=0.488, p=0.631 2-tailed). The regression slope does not differ significantly from 0 (t_23_=0.5, p=0.622 2-tailed). Visual inspection of the data, however, suggests that (aside from the rightmost point) the data suggests a bivariate uniform, rather than a bivariate normal, distribution. The RMA regression has a slope that is significantly steeper than both 0 and the slope of the OLS regression (t_22_=4.467, p≤0.0016), and an intercept significantly below both 0 and the intercept for the OLS regression (t_22_≥-2.337, p≈0.0002). More importantly, the intercept is significantly more negative than 0, as would be predicted by the brain lag effect (i.e. body size changes first). This is strongly suggestive of a dataset that is not bivariate normal, which in turn is *prima facie* evidence for a dataset that has grades.

To determine the optimal number of grades for a *k*-means cluster analysis, we plot a measure of the goodness of fit of the data to different numbers of clusters (in effect, the statistical ‘strain’ in the data) as a function of cluster number across a range of 2≤k≤7 clusters, with goodness of fit indexed as the F-statistic. Since the goodness of fit will at some point inevitably achieve an asymptotic value, we can identify the optimal number of clusters as the point on the X-axis corresponding to the slope transition point in the Y-axis. The latter is identified as the point on the Y-axis corresponding to 1/e^th^ down on the Y-axis from the asymptote.

Figure S2 plots the goodness of fit (indexed as the F-statistic) against number of clusters for the data in Fig. 1 (the plot of residual brain:body mass plotted against Perelman divergence dates). The F-values for all values of *k* are highly significant by a conventional analysis of variance (p<0.001). With an asymptotic value of F≈180, the optimal number of clusters corresponding to 1/e^th^ down from this is 4.5. We round this up to 5.


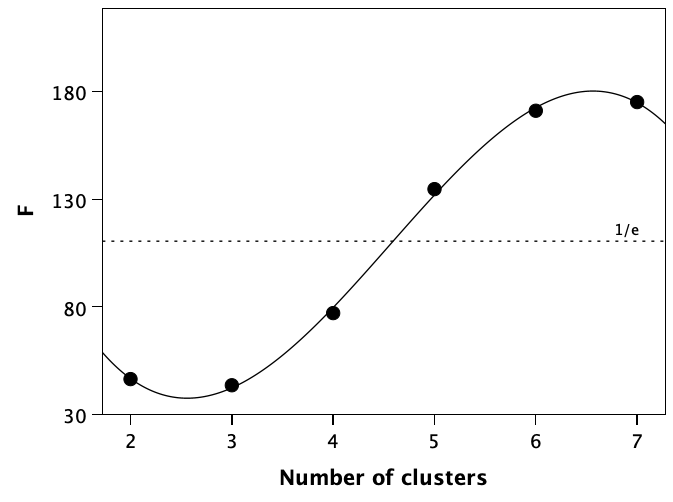


**S2 Figure.** **Optimal number of clusters.** Goodness of fit (indexed by F-value) for number of clusters in a *k*-means cluster analysis. The dotted line demarcates the point of inflexion (1/e^th^ down from the asymptote) that identifies (red vertical arrow) the optimal number of clusters that maximises fit while minimising partitioning of the data.


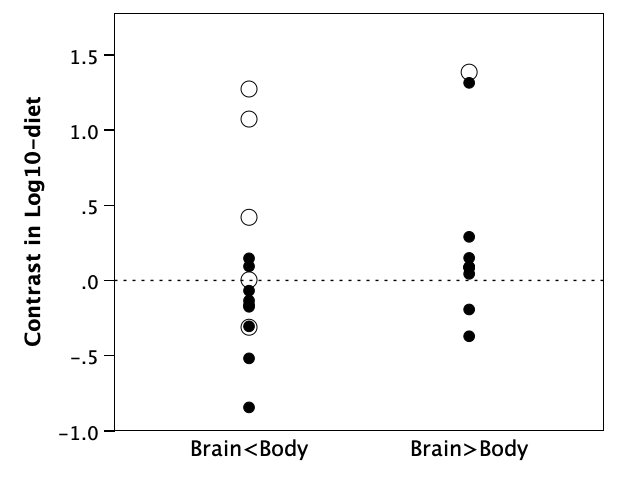


**S3 Figure.** **Contrasts in diet in relation to brain/body mass ratio.** Contrast in log_10_ percentage of fruit in diet for species where residual brain size is negative (brains are smaller than predicted for body size) versus those where it is positive (brains are larger than predicted). Filled symbols are haplorrhines; unfilled symbols are strepsirrhines.

**References**

Aiello, L. & Dunbar, R.I.M. (1993). Neocortex size, group size and the evolution of language. *Current Anthropology* 34: 184-193.

Cofran, Z. (2018). Brain size growth in wild and captive chimpanzees (*Pan troglodytes*). *American Journal of Primatology* 80: e22876.

Deaner, R.O. & Nunn, C.L. (1999). How quickly do brains catch up with bodies? A comparative method for detecting evolutionary lag. *Proceedings of the Royal Society, London*, 266B: 687-694.

De Miguel, C. & Henneberg, M. (2001). Variation in hominid brain size: how much is due to method? *Homo* 52: 3-58.

Dunbar, R.I.M. & Shultz, S. (2024). The social role of self-control. *BioRxiv* doi.org/10.1101/2020.10.26.354852

Heldstab, S. A., Kosonen, Z. K., Koski, S. E., Burkart, J. M., van Schaik, C. P., & Isler, K. (2016). Manipulation complexity in primates coevolved with brain size and terrestriality. *Scientific Reports* 6: 24528.

Powell, L. E., Isler, K. & Barton, R. A. (2017). Re-evaluating the link between brain size and behavioural ecology in primates. *Proceedings of the Royal Society, London*, 284B: 2017.1765.

Purvis, A. (1995). A composite estimate of primate phylogeny. *Philosophical Transactions of the Royal Society, London*, 348B: 405-421.

Pusey, A. E., Oehlert, G. W., Williams, J. M. & Goodall, J. (2005). Influence of ecological and social factors on body mass of wild chimpanzees. *International Journal of Primatology* 26: 3-31.

Will, M., Pablos, A. & Stock, J. T. (2017). Long-term patterns of body mass and stature evolution within the hominin lineage. *Royal Society Open Science* 4: 171339.
