# Supplementary material for: Evolutionary lags in the primate brain size/body size relationship revisited
Source: PLoS One. 2026 Jul 1;21(7):e0351073. doi: 10.1371/journal.pone.0351073 (PMC13322519; doi:10.1371/journal.pone.0351073)
Supplement: Attachment — Submitted filename: PLoS ONE reviews WITH RESPONSES.docx [file pone.0351073.s002.docx]

PONE-D-25-66844

**Evolutionary Lags in the Primate Brain Size/Body Size Relationship Revisited**

RESPONSE: Thank you for the opportunity to revise the MS in the light of the reviewers’ extremely helpful comments. Details of the changes made are given below. Changes to the text are indicated in red font.

Journal Requirements:

When submitting your revision, we need you to address these additional requirements.

1.Please ensure that your manuscript meets PLOS ONE's style requirements, including those for file naming. The PLOS ONE style templates can be found at

https://journals.plos.org/plosone/s/file?id=wjVg/PLOSOne_formatting_sample_main_body.pdf and

[https://journals.plos.org/plosone/s/file?id=ba62/PLOSOne_formatting_sample_title_authors_affiliations.pdf](https://track.editorialmanager.com/CL0/https:%2F%2Fjournals.plos.org%2Fplosone%2Fs%2Ffile%3Fid=ba62%2FPLOSOne_formatting_sample_title_authors_affiliations.pdf/1/010f019d3e3c0654-111d29e3-1f3c-494a-92db-79f05145ecce-000000/JIjkB2YJiQ9ClcUSG8QrKoboT7X1URfAkIUW7P1M4Uw=256)

RESPONSE: style requirements checked and implemented.

2. Please note that funding information should not appear in any section or other areas of your manuscript. We will only publish funding information present in the Funding Statement section of the online submission form. Please remove any funding-related text from the manuscript.

RESPONSE: funding information corrected.

3. We note that the grant information you provided in the ‘Funding Information’ and ‘Financial Disclosure’ sections do not match.

When you resubmit, please ensure that you provide the correct grant numbers for the awards you received for your study in the ‘Funding Information’ section.

RESPONSE: funding information corrected.

4. Please provide a complete Data Availability Statement in the submission form, ensuring you include all necessary access information or a reason for why you are unable to make your data freely accessible. If your research concerns only data provided within your submission, please write "All data are in the manuscript and/or supporting information files" as your Data Availability Statement.

RESPONSE: data availability statement clarified.

5. Please update your submission to use the PLOS LaTeX template. The template and more information on our requirements for LaTeX submissions can be found at <http://journals.plos.org/plosone/s/latex>.

RESPONSE: I don’t use Latex. The Author Instructions state that Word is acceptable.

6. If the reviewer comments include a recommendation to cite specific previously published works, please review and evaluate these publications to determine whether they are relevant and should be cited. There is no requirement to cite these works unless the editor has indicated otherwise.

RESPONSE: suggested new references all added.

7. Please review your reference list to ensure that it is complete and correct. If you have cited papers that have been retracted, please include the rationale for doing so in the manuscript text, or remove these references and replace them with relevant current references. Any changes to the reference list should be mentioned in the rebuttal letter that accompanies your revised manuscript. If you need to cite a retracted article, indicate the article’s retracted status in the References list and also include a citation and full reference for the retraction notice.

RESPONSE: referencing checked and formatted as required.

Reviewers' comments:

Reviewer's Responses to Questions

**Comments to the Author**

Reviewer #1: The paper “Evolutionary Lags in the Primate Brain Size/Body Size Relationship Revisited” provides thorough research on how brain size and body size are related in mammals. Author demonstrates a strong understanding of the topic and uses a clear, systematic approach. I have a few minor suggestions:

In the Methods section, it would help if the author explained better why RMA was chosen and what benefits it has compared to the more common OLS method.

RESPONSE: more detail has been added, with references.

In the Results section, Table 1 could be clearer if it included the standard errors and significance levels for the estimates.

RESPONSE: regression parameters with errors have been added to table, as requested.

Figure 1 is very interesting, but adding a legend to explain the symbols would make it easier to understand.

RESPONSE: The symbols differentiate the clusters revealed by the k-means cluster analysis. The details have now been added to the legend.

In the Discussion, the author should expand on how these findings relate to human evolution and what they mean for our understanding of brain development.
RESPONSE: a new paragraph commenting on implications for human evolution added to Discussion.

Overall, despite some small issues, this is a valuable piece of work that can be useful for other researchers studying evolution.

Reviewer #2: Dear Editors
This is my review of the manuscript Evolutionary Lags in the Primate Brain Size/Body Size Relationship Revisited.
The manuscript revisits the "brain lag hypothesis," which posits that during primate evolution, changes in brain size lagged behind changes in body size. Addressing a seminal 1999 analysis by Deaner & Nunn that found no evidence for this lag , the author re-analyzes their original dataset using updated molecular divergence dates and alternative statistical models (Reduced Major Axis regression and k-means clustering). The author concludes that a brain lag effect is indeed present. Furthermore, the study suggests that rather than merely catching up to a standard allometric baseline, certain primate lineages (and anthropoids in particular) overshot the regression line to occupy higher socio-cognitive end. The author attributes this to the cognitive demands of using large social groups as an anti-predator strategy, facilitated by energy savings from dietary shifts. The evolutionary trajectory of hominins is presented as a test case supporting this model.

Major Comments
The social brain is a fantastic hypothesis, but we know by now ECV is poor representation of the social brain circuitry, both in terms of functional neuroanatomy (Alcalá-López et al., 2018; Meshulam et al. 2025) and in evolutionary terms (Sansalone et al. 2023, Melchionna et al., 2025, Gutiérrez-Ibáñez). I feel the author should to the bare minimum, mention these studies and put a cautionary note about the use of brain volume to infer social cognition. This makes their arguments stronger, not weaker.

RESPONSE: Thanks for the references. I am very grateful to have these drawn to my attention: they are very relevant. They are now all added to the text.

Suggested citations:
Meshulam, L., Angelaki, D., Benson, J., McRoberts, I., Noel, J.-P., Arlandis, J., Bonacchi, N., Bougrova, K., Catarino, J. A., Cazettes, F., Crombie, D., DeWitt, E. E., Freitas-Silva, L., Laranjeira, I. C., Mainen, Z. F., Meijer, G. T., Rai, P., Raiser, G., Rau, F., … Witten, I. B. (2025). A brain-wide map of neural activity during complex behaviour. Nature, 645(8079), 177–191. https://doi.org/10.1038/s41586-025-09235-0

Melchionna, M., Castiglione, S., Girardi, G., Profico, A., Mondanaro, A., Sansalone, G., Chatar, N., Pérez Ramos, A., Fernández-Monescillo, M., Serio, C., Pandolfi, L., Dembitzer, J., di Febbraro, M., Caliendo, M. M., di Costanzo, A., Morvillo, L., Esposito, A., & Raia, P. (2025). Cortical areas associated to higher cognition drove primate brain evolution. Communications Biology, 8(1), 80. https://doi.org/10.1038/s42003-025-07505-1

Gutiérrez-Ibáñez, C., Němec, P., Paré, M., Wylie, D. R., & Lefebvre, L. (2025). How do big brains evolve? Trends in Ecology & Evolution, 0(0). https://doi.org/10.1016/j.tree.2025.03.008

Alcalá-López, D., Smallwood, J., Jefferies, E., van Overwalle, F., Vogeley, K., Mars, R. B., Turetsky, B. I., Laird, A. R., Fox, P. T., Eickhoff, S. B., & Bzdok, D. (2018). Computing the Social Brain Connectome Across Systems and States. Cerebral Cortex (New York, N.Y. : 1991), 28(7), 2207–2232. https://doi.org/10.1093/cercor/bhx121

Sansalone, G., Profico, A., Wroe, S., Allen, K., Ledogar, J., Ledogar, S., Mitchell, D. R., Mondanaro, A., Melchionna, M., Castiglione, S., Serio, C., & Raia, P. (2023). Homo sapiens and Neanderthals share high cerebral cortex integration into adulthood. Nature Ecology & Evolution, 7(1), 42–50. https://doi.org/10.1038/s41559-022-01933-6

The author purposedly restricted their analysis to the Stephan et al. (1981) dataset to perfectly mirror Deaner & Nunn’s original methodology and isolate the impact of the statistical/dating changes. While this is methodologically sound for a direct rebuttal, relying exclusively on a 40-year-old dataset limits the broader applicability of the findings. I feel the manuscript would vastly benefit from the inclusion of a supplementary analysis using a modern, comprehensive primate brain/body size dataset (even if it requires using carefully corrected ECV data). This would demonstrate that the brain lag effect is a robust biological reality, rather than a mathematical artifact specific to the 1981 sample.

RESPONSE: (1) Just because a database is 40 years old does not make it inaccurate! It was, after all, collated by German anatomists using histology, and German anatomists have been the best anatomists in the world for two centuries. In any case, there is no other comparable dataset: all the later datasets sample a smaller range of genera, and mostly do it with imaging data [which itself is subject to very considerable, well known measurement issues because of the difficulties associated with grey-scales]. The bottom line in the end is that none of the more recent primate databases offer a larger sample of primates than Stephan’s.

(2) If we are going to show why D&N were (partially) wrong, we really do need to show this using their original data. Using new datasets introduces new forms of uncertainty. This is already clear from the comparison of the two different dating phylogenies – the original early one and the more recent ones. This is why I give analyses for both sets: first to show that the original less accurate datings do give the same results and second to show that more recent, more accurate datings give a much more nuanced result that allows us to draw further conclusions.

The author uses a path analysis of phylogenetic contrasts to conclude a strict causal sequence: group size selects for brain size, which then selects for body. While the Sobel tests of mediation provide statistical support for this sequence, inferring evolutionary causality and directional selection purely from cross-sectional contrast correlations remains inherently risky. The language surrounding this finding should be softened to acknowledge the limitations of the data structure, or further theoretical justification should be provided for why reverse causality (e.g., spare energy from body size permitting larger groups) is impossible.

RESPONSE: This has already been done in a series of 4 published multivariate path analyses using different primate datasets with and without phylogenetic methods. All show that ‘spare energy from body size’ is a constraint [an energetic bottleneck], not a selection factor. What this points to is a crucial difference between **selection factors** and **consequential** **constraints** that are commonly confused by most comparative analyses. In other words, if you want to increase brain size [for any reason], then you need to solve the problem of how to fuel it. It does not mean that diet or nutrient acquisition is a selection factor for brain size! Correlations are not causes! The text has been reworded to make this clearer.

Also, using k-means clustering on the residuals to define five statistical grades is a mathematically elegant way to handle data heterogeneity. However, the author later asserts that these grades map closely onto socio-cognitive grades. To avoid the appearance of statistical circularity, the manuscript should briefly detail how these specific k-means clusters align with independent biological or behavioral metrics of socio-cognitive complexity. Providing a concrete example of the taxa within these clusters and their corresponding social behaviors would ground the statistical grades in biological reality.

RESPONSE: More detail and examples have been added to the text, and additional commentary added to Discussion as it ties in particularly nicely with the Melchionna et al. paper mentioned above.
