# Supplementary material for: Evolutionary lags in the primate brain size/body size relationship revisited
Source: PLoS One. 2026 Jul 1;21(7):e0351073. doi: 10.1371/journal.pone.0351073 (PMC13322519; doi:10.1371/journal.pone.0351073)
Supplement: Peer review history

# Peer review: Decision Letter 0

**DOI:** 10.1371/journal.pone.0351073.r001

**Authors:** Federica Spani

30 Mar 2026

PONE-D-25-66844

Evolutionary Lags in the Primate Brain Size/Body Size Relationship Revisited

PLOS One

Dear Dr. Dunbar,

Thank you for submitting your manuscript to PLOS ONE. After careful consideration, we feel that it has merit but does not fully meet PLOS ONE’s publication criteria as it currently stands. Therefore, we invite you to submit a revised version of the manuscript that addresses the points raised during the review process.

Please submit your revised manuscript by May 14 2026 11:59PM. If you will need more time than this to complete your revisions, please reply to this message or contact the journal office at plosone@plos.org. When you're ready to submit your revision, log on to https://www.editorialmanager.com/pone/ and select the 'Submissions Needing Revision' folder to locate your manuscript file.

Please include the following items when submitting your revised manuscript:

- A letter that responds to each point raised by the academic editor and reviewer(s). You should upload this letter as a separate file labeled 'Response to Reviewers'.
- A marked-up copy of your manuscript that highlights changes made to the original version. You should upload this as a separate file labeled 'Revised Manuscript with Track Changes'.
- An unmarked version of your revised paper without tracked changes. You should upload this as a separate file labeled 'Manuscript'.

If you would like to make changes to your financial disclosure, please include your updated statement in your cover letter. Guidelines for resubmitting your figure files are available below the reviewer comments at the end of this letter.

If applicable, we recommend that you deposit your laboratory protocols in protocols.io to enhance the reproducibility of your results. Protocols.io assigns your protocol its own identifier (DOI) so that it can be cited independently in the future. For instructions see: https://journals.plos.org/plosone/s/submission-guidelines#loc-laboratory-protocols. Additionally, PLOS ONE offers an option for publishing peer-reviewed Lab Protocol articles, which describe protocols hosted on protocols.io. Read more information on sharing protocols at https://plos.org/protocols?utm_medium=editorial-email&utm_source=authorletters&utm_campaign=protocols.

As the corresponding author, your ORCID iD is verified in the submission system and will appear in the published article. PLOS supports the use of ORCID, and we encourage all coauthors to register for an ORCID iD and use it as well. Please encourage your coauthors to verify their ORCID iD within the submission system before final acceptance, as unverified ORCID iDs will not appear in the published article. *Only*   the individual author can complete the verification step; PLOS staff *cannot*   verify ORCID iDs on behalf of authors.

We look forward to receiving your revised manuscript.

Kind regards,

Federica Spani, Ph.D

Academic Editor

PLOS One

Journal Requirements:

When submitting your revision, we need you to address these additional requirements.

1.Please ensure that your manuscript meets PLOS ONE's style requirements, including those for file naming. The PLOS ONE style templates can be found at

https://journals.plos.org/plosone/s/file?id=wjVg/PLOSOne_formatting_sample_main_body.pdf and

https://journals.plos.org/plosone/s/file?id=ba62/PLOSOne_formatting_sample_title_authors_affiliations.pdf

2. Please note that funding information should not appear in any section or other areas of your manuscript. We will only publish funding information present in the Funding Statement section of the online submission form. Please remove any funding-related text from the manuscript.

3. We note that the grant information you provided in the ‘Funding Information’ and ‘Financial Disclosure’ sections do not match.

When you resubmit, please ensure that you provide the correct grant numbers for the awards you received for your study in the ‘Funding Information’ section.

4. Please provide a complete Data Availability Statement in the submission form, ensuring you include all necessary access information or a reason for why you are unable to make your data freely accessible. If your research concerns only data provided within your submission, please write "All data are in the manuscript and/or supporting information files" as your Data Availability Statement.

5. Please update your submission to use the PLOS LaTeX template. The template and more information on our requirements for LaTeX submissions can be found at http://journals.plos.org/plosone/s/latex.

6. If the reviewer comments include a recommendation to cite specific previously published works, please review and evaluate these publications to determine whether they are relevant and should be cited. There is no requirement to cite these works unless the editor has indicated otherwise.

7. Please review your reference list to ensure that it is complete and correct. If you have cited papers that have been retracted, please include the rationale for doing so in the manuscript text, or remove these references and replace them with relevant current references. Any changes to the reference list should be mentioned in the rebuttal letter that accompanies your revised manuscript. If you need to cite a retracted article, indicate the article’s retracted status in the References list and also include a citation and full reference for the retraction notice.

[Note: HTML markup is below. Please do not edit.]

Reviewers' comments:

Reviewer's Responses to Questions

**Comments to the Author**

1. Is the manuscript technically sound, and do the data support the conclusions?

The manuscript must describe a technically sound piece of scientific research with data that supports the conclusions. Experiments must have been conducted rigorously, with appropriate controls, replication, and sample sizes. The conclusions must be drawn appropriately based on the data presented.

Reviewer #1: Yes

Reviewer #2: Yes

**********

2. Has the statistical analysis been performed appropriately and rigorously?

Reviewer #1: Yes

Reviewer #2: Yes

**********

3. Have the authors made all data underlying the findings in their manuscript fully available?

The PLOS Data policy requires authors to make all data underlying the findings described in their manuscript fully available without restriction, with rare exception (please refer to the Data Availability Statement in the manuscript PDF file). The data should be provided as part of the manuscript or its supporting information, or deposited to a public repository. For example, in addition to summary statistics, the data points behind means, medians and variance measures should be available. If there are restrictions on publicly sharing data—e.g. participant privacy or use of data from a third party—those must be specified.

Reviewer #1: Yes

Reviewer #2: Yes

**********

4. Is the manuscript presented in an intelligible fashion and written in standard English?

PLOS ONE does not copyedit accepted manuscripts, so the language in submitted articles must be clear, correct, and unambiguous. Any typographical or grammatical errors should be corrected at revision, so please note any specific errors here.

Reviewer #1: Yes

Reviewer #2: Yes

**********

5. Review Comments to the Author

Please use the space provided to explain your answers to the questions above. You may also include additional comments for the author, including concerns about dual publication, research ethics, or publication ethics. (Please upload your review as an attachment if it exceeds 20,000 characters)

Reviewer #1: The paper “Evolutionary Lags in the Primate Brain Size/Body Size Relationship Revisited” provides thorough research on how brain size and body size are related in mammals. Author demonstrates a strong understanding of the topic and uses a clear, systematic approach. I have a few minor suggestions:

In the Methods section, it would help if the author explained better why RMA was chosen and what benefits it has compared to the more common OLS method.

In the Results section, Table 1 could be clearer if it included the standard errors and significance levels for the estimates.

Figure 1 is very interesting, but adding a legend to explain the symbols would make it easier to understand.

In the Discussion, the author should expand on how these findings relate to human evolution and what they mean for our understanding of brain development.

Overall, despite some small issues, this is a valuable piece of work that can be useful for other researchers studying evolution.

Reviewer #2: Dear Editors

This is my review of the manuscript Evolutionary Lags in the Primate Brain Size/Body Size Relationship Revisited.

The manuscript revisits the "brain lag hypothesis," which posits that during primate evolution, changes in brain size lagged behind changes in body size. Addressing a seminal 1999 analysis by Deaner & Nunn that found no evidence for this lag , the author re-analyzes their original dataset using updated molecular divergence dates and alternative statistical models (Reduced Major Axis regression and k-means clustering). The author concludes that a brain lag effect is indeed present. Furthermore, the study suggests that rather than merely catching up to a standard allometric baseline, certain primate lineages (and anthropoids in particular) overshot the regression line to occupy higher socio-cognitive end. The author attributes this to the cognitive demands of using large social groups as an anti-predator strategy, facilitated by energy savings from dietary shifts. The evolutionary trajectory of hominins is presented as a test case supporting this model.

Major Comments

The social brain is a fantastic hypothesis, but we know by now ECV is poor representation of the social brain circuitry, both in terms of functional neuroanatomy (Alcalá-López et al., 2018; Meshulam et al. 2025) and in evolutionary terms (Sansalone et al. 2023, Melchionna et al., 2025, Gutiérrez-Ibáñez). I feel the author should to the bare minimum, mention these studies and put a cautionary note about the use of brain volume to infer social cognition. This makes their arguments stronger, not weaker.

Suggested citations:

Meshulam, L., Angelaki, D., Benson, J., McRoberts, I., Noel, J.-P., Arlandis, J., Bonacchi, N., Bougrova, K., Catarino, J. A., Cazettes, F., Crombie, D., DeWitt, E. E., Freitas-Silva, L., Laranjeira, I. C., Mainen, Z. F., Meijer, G. T., Rai, P., Raiser, G., Rau, F., … Witten, I. B. (2025). A brain-wide map of neural activity during complex behaviour. Nature, 645(8079), 177–191. https://doi.org/10.1038/s41586-025-09235-0

Melchionna, M., Castiglione, S., Girardi, G., Profico, A., Mondanaro, A., Sansalone, G., Chatar, N., Pérez Ramos, A., Fernández-Monescillo, M., Serio, C., Pandolfi, L., Dembitzer, J., di Febbraro, M., Caliendo, M. M., di Costanzo, A., Morvillo, L., Esposito, A., & Raia, P. (2025). Cortical areas associated to higher cognition drove primate brain evolution. Communications Biology, 8(1), 80. https://doi.org/10.1038/s42003-025-07505-1

Gutiérrez-Ibáñez, C., Němec, P., Paré, M., Wylie, D. R., & Lefebvre, L. (2025). How do big brains evolve? Trends in Ecology & Evolution, 0(0). https://doi.org/10.1016/j.tree.2025.03.008

Alcalá-López, D., Smallwood, J., Jefferies, E., van Overwalle, F., Vogeley, K., Mars, R. B., Turetsky, B. I., Laird, A. R., Fox, P. T., Eickhoff, S. B., & Bzdok, D. (2018). Computing the Social Brain Connectome Across Systems and States. Cerebral Cortex (New York, N.Y. : 1991), 28(7), 2207–2232. https://doi.org/10.1093/cercor/bhx121

Sansalone, G., Profico, A., Wroe, S., Allen, K., Ledogar, J., Ledogar, S., Mitchell, D. R., Mondanaro, A., Melchionna, M., Castiglione, S., Serio, C., & Raia, P. (2023). Homo sapiens and Neanderthals share high cerebral cortex integration into adulthood. Nature Ecology & Evolution, 7(1), 42–50. https://doi.org/10.1038/s41559-022-01933-6

The author purposedly restricted their analysis to the Stephan et al. (1981) dataset to perfectly mirror Deaner & Nunn’s original methodology and isolate the impact of the statistical/dating changes. While this is methodologically sound for a direct rebuttal, relying exclusively on a 40-year-old dataset limits the broader applicability of the findings. I feel the manuscript would vastly benefit from the inclusion of a supplementary analysis using a modern, comprehensive primate brain/body size dataset (even if it requires using carefully corrected ECV data). This would demonstrate that the brain lag effect is a robust biological reality, rather than a mathematical artifact specific to the 1981 sample.

The author uses a path analysis of phylogenetic contrasts to conclude a strict causal sequence: group size selects for brain size, which then selects for body. While the Sobel tests of mediation provide statistical support for this sequence, inferring evolutionary causality and directional selection purely from cross-sectional contrast correlations remains inherently risky. The language surrounding this finding should be softened to acknowledge the limitations of the data structure, or further theoretical justification should be provided for why reverse causality (e.g., spare energy from body size permitting larger groups) is impossible.

Also, using k-means clustering on the residuals to define five statistical grades is a mathematically elegant way to handle data heterogeneity. However, the author later asserts that these grades map closely onto socio-cognitive grades. To avoid the appearance of statistical circularity, the manuscript should briefly detail how these specific k-means clusters align with independent biological or behavioral metrics of socio-cognitive complexity. Providing a concrete example of the taxa within these clusters and their corresponding social behaviors would ground the statistical grades in biological reality.

**********

6. PLOS authors have the option to publish the peer review history of their article (what does this mean?). If published, this will include your full peer review and any attached files.

If you choose “no”, your identity will remain anonymous but your review may still be made public.

**Do you want your identity to be public for this peer review?**   For information about this choice, including consent withdrawal, please see our Privacy Policy.

Reviewer #1: No

Reviewer #2: No

**********

[NOTE: If reviewer comments were submitted as an attachment file, they will be attached to this email and accessible via the submission site. Please log into your account, locate the manuscript record, and check for the action link "View Attachments". If this link does not appear, there are no attachment files.]

To ensure your figures meet our technical requirements, please review our figure guidelines: https://journals.plos.org/plosone/s/figures

You may also use PLOS’s free figure tool, NAAS, to help you prepare publication quality figures: https://journals.plos.org/plosone/s/figures#loc-tools-for-figure-preparation.

NAAS will assess whether your figures meet our technical requirements by comparing each figure against our figure specifications.

# Author comment: Author response to Decision Letter 1

**DOI:** 10.1371/journal.pone.0351073.r002

23 Apr 2026

PONE-D-25-66844

Evolutionary Lags in the Primate Brain Size/Body Size Relationship Revisited

RESPONSE: Thank you for the opportunity to revise the MS in the light of the reviewers’ extremely helpful comments. Details of the changes made are given below. Changes to the text are indicated in red font.

Journal Requirements:

When submitting your revision, we need you to address these additional requirements.

1.Please ensure that your manuscript meets PLOS ONE's style requirements, including those for file naming. The PLOS ONE style templates can be found at

https://journals.plos.org/plosone/s/file?id=wjVg/PLOSOne_formatting_sample_main_body.pdf and

https://journals.plos.org/plosone/s/file?id=ba62/PLOSOne_formatting_sample_title_authors_affiliations.pdf

RESPONSE: style requirements checked and implemented.

2. Please note that funding information should not appear in any section or other areas of your manuscript. We will only publish funding information present in the Funding Statement section of the online submission form. Please remove any funding-related text from the manuscript.

RESPONSE: funding information corrected.

3. We note that the grant information you provided in the ‘Funding Information’ and ‘Financial Disclosure’ sections do not match.

When you resubmit, please ensure that you provide the correct grant numbers for the awards you received for your study in the ‘Funding Information’ section.

RESPONSE: funding information corrected.

4. Please provide a complete Data Availability Statement in the submission form, ensuring you include all necessary access information or a reason for why you are unable to make your data freely accessible. If your research concerns only data provided within your submission, please write "All data are in the manuscript and/or supporting information files" as your Data Availability Statement.

RESPONSE: data availability statement clarified.

5. Please update your submission to use the PLOS LaTeX template. The template and more information on our requirements for LaTeX submissions can be found at http://journals.plos.org/plosone/s/latex.

RESPONSE: I don’t use Latex. The Author Instructions state that Word is acceptable.

6. If the reviewer comments include a recommendation to cite specific previously published works, please review and evaluate these publications to determine whether they are relevant and should be cited. There is no requirement to cite these works unless the editor has indicated otherwise.

RESPONSE: suggested new references all added.

7. Please review your reference list to ensure that it is complete and correct. If you have cited papers that have been retracted, please include the rationale for doing so in the manuscript text, or remove these references and replace them with relevant current references. Any changes to the reference list should be mentioned in the rebuttal letter that accompanies your revised manuscript. If you need to cite a retracted article, indicate the article’s retracted status in the References list and also include a citation and full reference for the retraction notice.

RESPONSE: referencing checked and formatted as required.

Reviewers' comments:

Reviewer's Responses to Questions

Comments to the Author

Reviewer #1: The paper “Evolutionary Lags in the Primate Brain Size/Body Size Relationship Revisited” provides thorough research on how brain size and body size are related in mammals. Author demonstrates a strong understanding of the topic and uses a clear, systematic approach. I have a few minor suggestions:

In the Methods section, it would help if the author explained better why RMA was chosen and what benefits it has compared to the more common OLS method.

RESPONSE: more detail has been added, with references.

In the Results section, Table 1 could be clearer if it included the standard errors and significance levels for the estimates.

RESPONSE: regression parameters with errors have been added to table, as requested.

Figure 1 is very interesting, but adding a legend to explain the symbols would make it easier to understand.

RESPONSE: The symbols differentiate the clusters revealed by the k-means cluster analysis. The details have now been added to the legend.

In the Discussion, the author should expand on how these findings relate to human evolution and what they mean for our understanding of brain development.

RESPONSE: a new paragraph commenting on implications for human evolution added to Discussion.

Overall, despite some small issues, this is a valuable piece of work that can be useful for other researchers studying evolution.

Reviewer #2: Dear Editors

This is my review of the manuscript Evolutionary Lags in the Primate Brain Size/Body Size Relationship Revisited.

The manuscript revisits the "brain lag hypothesis," which posits that during primate evolution, changes in brain size lagged behind changes in body size. Addressing a seminal 1999 analysis by Deaner & Nunn that found no evidence for this lag , the author re-analyzes their original dataset using updated molecular divergence dates and alternative statistical models (Reduced Major Axis regression and k-means clustering). The author concludes that a brain lag effect is indeed present. Furthermore, the study suggests that rather than merely catching up to a standard allometric baseline, certain primate lineages (and anthropoids in particular) overshot the regression line to occupy higher socio-cognitive end. The author attributes this to the cognitive demands of using large social groups as an anti-predator strategy, facilitated by energy savings from dietary shifts. The evolutionary trajectory of hominins is presented as a test case supporting this model.

Major Comments

The social brain is a fantastic hypothesis, but we know by now ECV is poor representation of the social brain circuitry, both in terms of functional neuroanatomy (Alcalá-López et al., 2018; Meshulam et al. 2025) and in evolutionary terms (Sansalone et al. 2023, Melchionna et al., 2025, Gutiérrez-Ibáñez). I feel the author should to the bare minimum, mention these studies and put a cautionary note about the use of brain volume to infer social cognition. This makes their arguments stronger, not weaker.

RESPONSE: Thanks for the references. I am very grateful to have these drawn to my attention: they are very relevant. They are now all added to the text.

Suggested citations:

Meshulam, L., Angelaki, D., Benson, J., McRoberts, I., Noel, J.-P., Arlandis, J., Bonacchi, N., Bougrova, K., Catarino, J. A., Cazettes, F., Crombie, D., DeWitt, E. E., Freitas-Silva, L., Laranjeira, I. C., Mainen, Z. F., Meijer, G. T., Rai, P., Raiser, G., Rau, F., … Witten, I. B. (2025). A brain-wide map of neural activity during complex behaviour. Nature, 645(8079), 177–191. https://doi.org/10.1038/s41586-025-09235-0

Melchionna, M., Castiglione, S., Girardi, G., Profico, A., Mondanaro, A., Sansalone, G., Chatar, N., Pérez Ramos, A., Fernández-Monescillo, M., Serio, C., Pandolfi, L., Dembitzer, J., di Febbraro, M., Caliendo, M. M., di Costanzo, A., Morvillo, L., Esposito, A., & Raia, P. (2025). Cortical areas associated to higher cognition drove primate brain evolution. Communications Biology, 8(1), 80. https://doi.org/10.1038/s42003-025-07505-1

Gutiérrez-Ibáñez, C., Němec, P., Paré, M., Wylie, D. R., & Lefebvre, L. (2025). How do big brains evolve? Trends in Ecology & Evolution, 0(0). https://doi.org/10.1016/j.tree.2025.03.008

Alcalá-López, D., Smallwood, J., Jefferies, E., van Overwalle, F., Vogeley, K., Mars, R. B., Turetsky, B. I., Laird, A. R., Fox, P. T., Eickhoff, S. B., & Bzdok, D. (2018). Computing the Social Brain Connectome Across Systems and States. Cerebral Cortex (New York, N.Y. : 1991), 28(7), 2207–2232. https://doi.org/10.1093/cercor/bhx121

Sansalone, G., Profico, A., Wroe, S., Allen, K., Ledogar, J., Ledogar, S., Mitchell, D. R., Mondanaro, A., Melchionna, M., Castiglione, S., Serio, C., & Raia, P. (2023). Homo sapiens and Neanderthals share high cerebral cortex integration into adulthood. Nature Ecology & Evolution, 7(1), 42–50. https://doi.org/10.1038/s41559-022-01933-6

The author purposedly restricted their analysis to the Stephan et al. (1981) dataset to perfectly mirror Deaner & Nunn’s original methodology and isolate the impact of the statistical/dating changes. While this is methodologically sound for a direct rebuttal, relying exclusively on a 40-year-old dataset limits the broader applicability of the findings. I feel the manuscript would vastly benefit from the inclusion of a supplementary analysis using a modern, comprehensive primate brain/body size dataset (even if it requires using carefully corrected ECV data). This would demonstrate that the brain lag effect is a robust biological reality, rather than a mathematical artifact specific to the 1981 sample.

RESPONSE: (1) Just because a database is 40 years old does not make it inaccurate! It was, after all, collated by German anatomists using histology, and German anatomists have been the best anatomists in the world for two centuries. In any case, there is no other comparable dataset: all the later datasets sample a smaller range of genera, and mostly do it with imaging data [which itself is subject to very considerable, well known measurement issues because of the difficulties associated with grey-scales]. The bottom line in the end is that none of the more recent primate databases offer a larger sample of primates than Stephan’s.

(2) If we are going to show why D&N were (partially) wrong, we really do need to show this using their original data. Using new datasets introduces new forms of uncertainty. This is already clear from the comparison of the two different dating phylogenies – the original early one and the more recent ones. This is why I give analyses for both sets: first to show that the original less accurate datings do give the same results and second to show that more recent, more accurate datings give a much more nuanced result that allows us to draw further conclusions.

The author uses a path analysis of phylogenetic contrasts to conclude a strict causal sequence: group size selects for brain size, which then selects for body. While the Sobel tests of mediation provide statistical support for this sequence, inferring evolutionary causality and directional selection purely from cross-sectional contrast correlations remains inherently risky. The language surrounding this finding should be softened to acknowledge the limitations of the data structure, or further theoretical justification should be provided for why reverse causality (e.g., spare energy from body size permitting larger groups) is impossible.

RESPONSE: This has already been done in a series of 4 published multivariate path analyses using different primate datasets with and without phylogenetic methods. All show that ‘spare energy from body size’ is a constraint [an energetic bottleneck], not a selection factor. What this points to is a crucial difference between selection factors and consequential constraints that are commonly confused by most comparative analyses. In other words, if you want to increase brain size [for any reason], then you need to solve the problem of how to fuel it. It does not mean that diet or nutrient acquisition is a selection factor for brain size! Correlations are not causes! The text has been reworded to make this clearer.

Also, using k-means clustering on the residuals to define five statistical grades is a mathematically elegant way to handle data heterogeneity. However, the author later asserts that these grades map closely onto socio-cognitive grades. To avoid the appearance of statistical circularity, the manuscript should briefly detail how these specific k-means clusters align with independent biological or behavioral metrics of socio-cognitive complexity. Providing a concrete example of the taxa within these clusters and their corresponding social behaviors would ground the statistical grades in biological reality.

RESPONSE: More detail and examples have been added to the text, and additional commentary added to Discussion as it ties in particularly nicely with the Melchionna et al. paper mentioned above.

Attachment Submitted filename: PLoS ONE reviews WITH RESPONSES.docx

# Editor report: Decision Letter 1

**DOI:** 10.1371/journal.pone.0351073.r003

**Authors:** Federica Spani

21 May 2026

Evolutionary Lags in the Primate Brain Size/Body Size Relationship Revisited

PONE-D-25-66844R1

Dear Dr. Dunbar,

We’re pleased to inform you that your manuscript has been judged scientifically suitable for publication and will be formally accepted for publication once it meets all outstanding technical requirements.

Within one week, you’ll receive an e-mail detailing the required amendments. When these have been addressed, you’ll receive a formal acceptance letter and your manuscript will be scheduled for publication.

An invoice will be generated when your article is formally accepted. Please note, if your institution has a publishing partnership with PLOS and your article meets the relevant criteria, all or part of your publication costs will be covered. Please make sure your user information is up-to-date by logging into Editorial Manager at Editorial Manager® and clicking the ‘Update My Information' link at the top of the page. For questions related to billing, please contact billing support.

If your institution or institutions have a press office, please notify them about your upcoming paper to help maximize its impact. If they’ll be preparing press materials, please inform our press team as soon as possible -- no later than 48 hours after receiving the formal acceptance. Your manuscript will remain under strict press embargo until 2 pm Eastern Time on the date of publication. For more information, please contact onepress@plos.org.

Kind regards,

Federica Spani, Ph.D

Academic Editor

PLOS One

Additional Editor Comments (optional):

Reviewers' comments:

# Editor report: Acceptance letter

**DOI:** 10.1371/journal.pone.0351073.r004

**Authors:** Federica Spani

PONE-D-25-66844R1

PLOS One

Dear Dr. Dunbar,

I'm pleased to inform you that your manuscript has been deemed suitable for publication in PLOS One. Congratulations! Your manuscript is now being handed over to our production team.

At this stage, our production department will prepare your paper for publication. This includes ensuring the following:

* All references, tables, and figures are properly cited

* All relevant supporting information is included in the manuscript submission,

* There are no issues that prevent the paper from being properly typeset

You will receive further instructions from the production team, including instructions on how to review your proof when it is ready. Please keep in mind that we are working through a large volume of accepted articles, so please give us a few days to review your paper and let you know the next and final steps.

Lastly, if your institution or institutions have a press office, please let them know about your upcoming paper now to help maximize its impact. If they'll be preparing press materials, please inform our press team within the next 48 hours. Your manuscript will remain under strict press embargo until 2 pm Eastern Time on the date of publication. For more information, please contact onepress@plos.org.

You will receive an invoice from PLOS for your publication fee after your manuscript has reached the completed accept phase. If you receive an email requesting payment before acceptance or for any other service, this may be a phishing scheme. Learn how to identify phishing emails and protect your accounts at https://explore.plos.org/phishing.

If we can help with anything else, please email us at customercare@plos.org.

Thank you for submitting your work to PLOS ONE and supporting open access.

Kind regards,

PLOS ONE Editorial Office Staff

on behalf of

Dr. Federica Spani

Academic Editor

PLOS One
